# Integrated DIA proteomics and lipidomics analysis on non-small cell lung cancer patients with TCM syndromes

**DOI:** 10.1186/s13020-021-00535-x

**Published:** 2021-11-27

**Authors:** Song Cang, Ran Liu, Wei Jin, Qi Tang, Wanjun Li, Kunqian Mu, Pengfei Jin, Kaishun Bi, Qing Li

**Affiliations:** 1 School of Pharmacy, National and Local Joint Engineering Laboratory for Key Technology of Chinese Material Medica Quality Control, Shenyang Pharmaceutical University, 103 Wenhua Road, Shenyang, 110016 China; 2 School of Applied Chemistry and Biological Technology, Shenzhen Polytechnic, 7098 Lau sin Avenue, Shenzhen, 518000 China; 3 Department of Chinese Medicine, National Cancer Center/National Clinical Research Center for Cancer/Cancer Hospital, Chinese Academy of Medical Sciences and Peking Union Medical College, No. 17, Panjiayuan Nanli, Chaoyang, Beijing, 100021 China; 4 Department of Pharmaceutical Science, Beijing Key Laboratory of Assessment of Clinical Drugs Risk and Individual Application, Beijing Hospital, National Center of Gerontology, Institute of Geriatric Medicine, Chinese Academy of Medical Science, No. 1 Dahua Road, Dong Dan, Beijing, 100730 China

**Keywords:** Non-small cell lung cancer, Traditional Chinese medicine syndromes, Syndrome differentiation, Lipidomics, Proteomics

## Abstract

**Background:**

Lung cancer remains the leading cause of mortality from malignant tumors, non-small cell lung cancer (NSCLC) accounts for the majority of lung cancer cases, and individualized diagnosis and treatment is an effective trend. The individual characteristics of different traditional Chinese medicine (TCM) syndromes of NSCLC patients may be revealed by highly specific molecular profiles.

**Methods:**

In this study, 10 NSCLC patients with Qi deficiency and Yin deficiency (QDYD) syndrome and 10 patients with Qi deficiency of lung-spleen (QDLS) syndrome in TNM stage III-IV as well as 10 healthy volunteers were enrolled. Aiming at the varied syndromes of NSCLC patients with “Yin deficiency” as the main difference, a proteomics research based on data-independent acquisition (DIA) was developed. Of the dysregulated proteins in NSCLC patients, lipid metabolism was significantly enriched. Thereafter, nontargeted lipidomics research based on UPLC-Q-TOF/MS was performed in 16 patients, with 8 individuals randomly selected from each syndrome group. Furthermore, the considerably different characteristics between the syndromes and pathological mechanisms of NSCLC were screened by statistical and biological integrations of proteomics and lipidomics and the differential metabolic pathways of the two similar syndromes were further explored. Besides, lipids biomarkers were verified by a clinically used anticancer Chinese medicine, and the level of key differential proteins in the two syndromes was also validated using ELISA.

**Results:**

The results showed that glycerophospholipid metabolism, sphingolipid metabolism, glycolipid metabolism, and primary bile acid biosynthesis were altered in NSCLC patients and that glycerophospholipid metabolism was significantly changed between the two syndromes in lipidomics analysis. Among the proteins and lipids, ALDOC and lysophosphatidylcholine (LPCs) were revealed to have a strong relationship by statistical and biological integration analysis, and could effectively distinguish QDLS and QDYD syndromes. Notably, the patients with different syndromes had the most typical metabolic patterns in glycerophospholipid metabolism and glycolysis, reflecting the differences in the syndromes dominated by “Yin deficiency”.

**Conclusions:**

ALDOC and LPCs could be employed for the differentiation of NSCLC patients with QDLS and QDYD syndromes, and “Yin deficiency” might be associated with glycerophospholipid metabolism and glycolysis pathway. The results provided a theoretical basis for “Syndrome differentiation” in TCM diagnosis. Moreover, the developed integrated strategy could also provide a reference for individualized diagnosis and treatment of other diseases.

**Supplementary Information:**

The online version contains supplementary material available at 10.1186/s13020-021-00535-x.

## Background

Lung cancer accounts for most cancer deaths worldwide with increasing incidence [1]. Approximately 85% of lung cancer cases are identified as non-small cell lung cancer (NSCLC), and approximately two-thirds of lung cancer cases are recognized only in advanced stages and have a poor prognosis. Traditional Chinese medicine (TCM) is a time-honored practice and is gaining in popularity in Asia, where it plays an important role in the treatment of lung cancer, as well as other countries in the West. Evidence on improving quality of life, prolonging survival time and reducing chemotherapy induced toxicity has demonstrated the effectiveness of TCM, especially for patients with advanced-stage lung cancer [2]. As the most prominent advantage of TCM, individualized treatment could be implemented based on syndrome differentiation, consistent with the idea of personalized medicine and precision medicine. TCM syndrome is now generally accepted as a reflection of multisystem and multiorgan functional impairment. Through the description of TCM syndrome, the subjective feeling of patients as well as clinical manifestations of diseases can be reflected, and the essence of pathological changes at certain stages of a disease can be revealed [3].

**Fig. 1:**
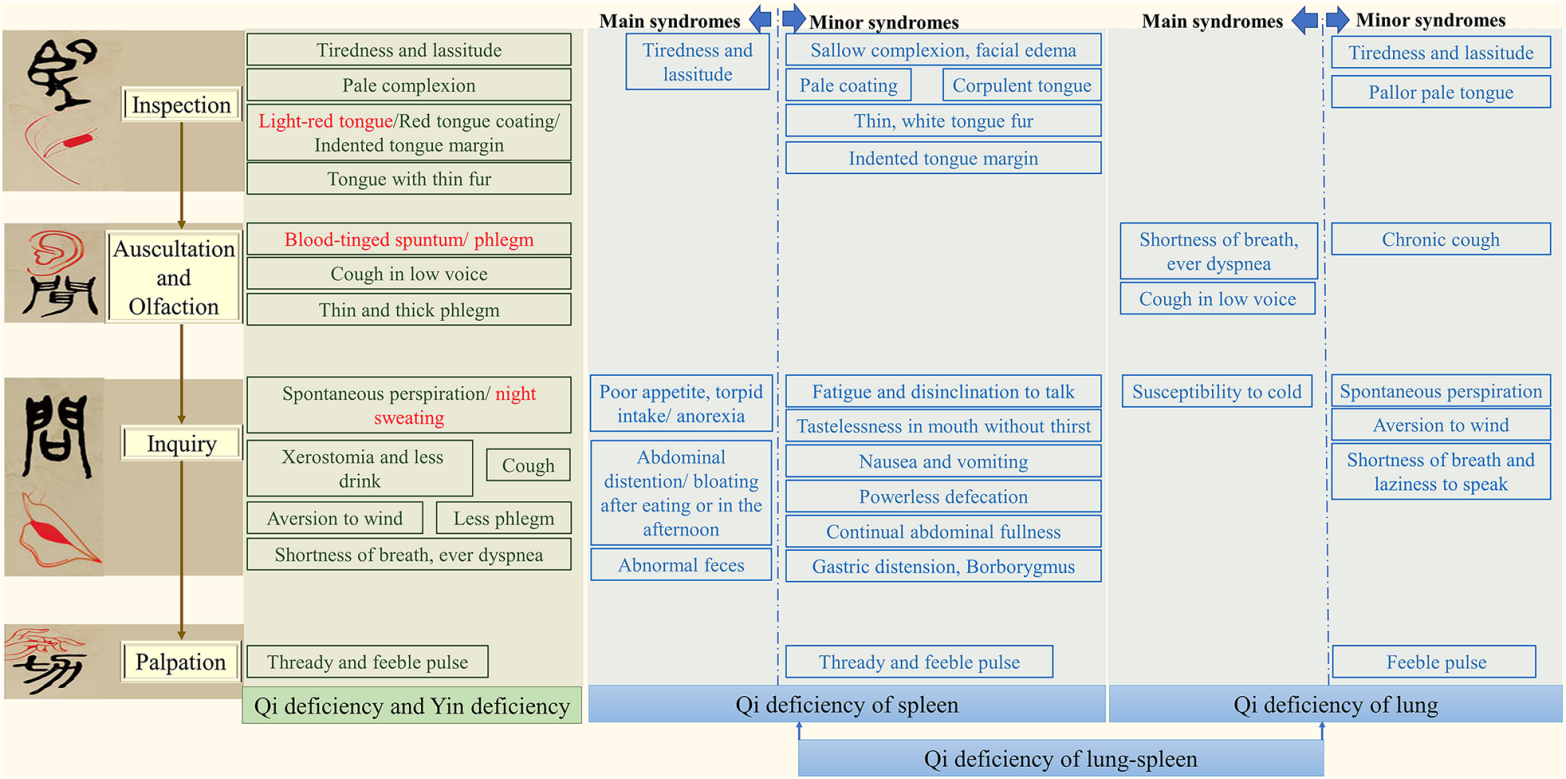
The detailed diagnostic procedure based on four examinations in TCM syndrome type diagnosis and differentiation

Clinical practice has shown that lung cancer is a chronic consumptive disease, of which most are deficiency syndromes. Among patients with advanced NSCLC, Qi deficiency of the lung-spleen (QDLS) and Qi deficiency and Yin deficiency (QDYD) in TCM syndromes are commonly diagnosed. Both syndromes have Qi deficiency syndrome. From the theory of TCM, the concept of “Qi” is the vital energy of the human body. It helps to maintain blood circulation and fight disease [3]. The occurrence of lung cancer is closely related to the deficiency of vital energy and the invasion of pathogenic factors. The loss of vital energy is mainly caused by aging, chronic illness, eating disorders, and overwork, which lead to the disturbance of diffusion and downbearing of the qi of the lung, and the inability to transport the body fluid. As a result, the blood circulation is blocked, body fluid retained internally, and the phlegm, stasis and toxin are generated over time. Then the lung is cemented to form tumors. After the onset of the disease, patients will appear diverse clinical manifestations due to the personal physique and acquired reasons, and thus be identified as different syndromes (Fig. 1). Although both syndromes have Qi deficiency syndrome, however, it was reported that the CYFRA21-1 levels in QDYD patients were significantly higher than those in QDLS patients [4]. Meanwhile, there are certain differences in symptoms that could reflect the prominent feature of these two syndromes in NSCLC patients, it could be speculated that there are discrepancies in tumor metabolism between the two syndromes dominated cause by “Yin deficiency”, which requires further exploration. At present, the different syndromes in TCM are identified by manifestations, lacking objective basis and laboratory markers. In addition, treatment should also vary according to the patient. Taken together, a quantitative model is necessary to evaluate TCM syndrome of NSCLC that can also avoid the subjective error caused by the experience of TCM practitioners.

Omics approaches—emerging as systems biology analysis at a global level—could effectively facilitate the differentiation of syndromes [5]. Proteomics and lipidomics were proposed as the two techniques that could unravel the essence of complex etiologies from different viewpoints [6, 7]. As an omics approach that could provide a real and microscopic perspective to view the molecular level of patients at a specific time, proteomics could be combined with lipidomics to accelerate the discovery of robust biomarkers and investigate the interrelationship between lipid and protein changes [8]. This integration enables comprehensive assessment of changes between different syndromes in lung cancer and reveals relevant pathological mechanisms. Lipids can reflect the metabolic results of the human body, and joint verification between proteins and lipids is more reasonable and important than verification between proteins alone. Thus, all of the separate efforts, the lipid or protein feature discrepancies of different syndromes of NSCLC patients who could be helped for proper diagnosis by TCM doctors have not been explored, emphasizing the significance of the construction of omics approaches in this field.

In summary, in this paper, a specific data mine for disease syndrome type could be built by 1. Applying DIA proteomics technology to analyze protein compositions of different syndromes, and using bioinformatics and statistical analysis to screen protein expression profiles, then dissecting the abnormal protein signaling pathways, 2. Applying UPLC-Q-TOF/MS lipidomics technology to analyze lipids compositions of different syndromes. The rationality of lipid biomarkers was further corroborated by Kangai injection, a commonly used anticancer Chinese medicine in the clinic, composed of Ginseng radix et rhizoma, Astragali radix and oxymatrine. In addition, the differential proteins and lipid species that distinguish lung cancer patients from healthy people or the two syndromes were analyzed in depth by statistical and biological integration analysis, which mutually verified the phenotypic differences of varying types of NSCLC. Moreover, the key differentially expressed protein between the two syndromes was also validated using ELISA. The results of molecular features and data mining based on omics for TCM syndrome differentiation in NSCLC provide a meaningful reference for individualized clinical diagnosis. Different metabolic patterns were also used to reflect the discrepancy of syndromes. The strategy of integrating TCM and modern medicine is the transformation from experience and concept of TCM to biochemical and biological meaning, which could contribute greatly to clinical TCM diagnosis and treatment of cancer.

## Material and methods

### Patients

This study was approved by the Ethics Committee of Urumqi Hospital of Chinese Medicine. A total of 20 patients with pathologically confirmed NSCLC and differentiated QDLS or QDYD syndromes were enrolled in Urumqi Hospital of Chinese Medicine between 2017 and 2019. There were 10 patients aged 43–86 with a mean age of 66 in the QDLS group, and 10 patients aged 51–84 with a median age of 74 in the QDYD group. Pretreatment examination included CT and MRI, and the staging system of TNM was adapted for clinical staging. Except for one patient in the QDLS group with stage IIIc disease, all other patients in the QDLS and QDYD groups were had stage IV NSCLC. A total of 10 healthy volunteers who were aged 53–78 with a mean age of 70 were enrolled in the same hospital at the same time (there was no significant difference in age between each group). The detailed characteristics of the subjects were described in Additional file 1: Table S1. They were all enrolled in the proteomics analysis. All patients and healthy volunteers were ethnically Han. The study was conducted in accordance with the Declaration of Helsinki, and was approved by local institutional review boards. Each participant signed an informed consent form.

For analysis validation, 8 patients were selected randomly from each TCM syndrome group in the proteomics study and were given Kangai injection for therapy. Subjects in each treatment group were injected intravenously with the equivalent of 40 ml of Kangai injection every day for 30 days. To make the number of samples consistent before and after treatment, 8 patients from each group who were given Kangai injection after blood collection were selected for lipidomics analysis.

To reduce the subjectivity in TCM syndrome type diagnosis and differentiation, the QDLS and QDYD syndromes of NSCLC were differentiated according to diagnosis from three TCM experts under the guideline of the national standard “The diagnosis and treatment program of lung cancer issued by the Administration of Traditional Chinese Medicine”. Each patient received a personal diagnosis based on TCM syndrome, which was a characteristic phenotype of identifiable manifestations gleaned from the following four examinations. First, called “inspection” (Wang in Chinese of fourth tone), the patient’s skin complexion and physique and tongue condition were inspected. Then, called “auscultation and olfaction” (Wen in Chinese of second tone), the patient’s voice was heard to see if there were any breathing problems, cough or phlegm, and the patient’s body odors were sniffed. Next, called “inquiry” (Wen in Chinese of fourth tone), the patient answered the questions from practitioners about their feelings overall, such as feeling hot or cold, whether they were sweating or were thirsty, how did stools look, etc. Finally, called “palpation” (Qie in Chinese of fourth tone), the TCM practitioner palpated the patient’s wrist to feel the quality of the pulse. The detailed diagnostic procedure is described in Fig. 1.

### Data independent acquisition (DIA)-based proteomic analysis

#### Samples preparation for proteomics

Lysis buffer [1% SDS, 8 M urea, 1 ×  Protease Inhibitor Cocktail (Roche Ltd., Basel, Switzerland)] was added into the plasma samples, vibrated and milling for 400 s for three times. The samples were then lysed on ice for 30 min and centrifuged at 15,000 rpm for 15 min at 4 ℃. The supernatant was collected and transferred to a new Eppendorf tube. 100 μg of protein per condition measured by BCA Protein Assay Kit was transferred into a new Eppendorf tube and the final volume was adjusted to 100 μL with 8 M urea. 2 μL of 0.5 M TCEP was added and the sample was incubated at 37 ℃ for 1 h, and then 4 μL of 1 M iodoacetamide was added to the sample and the incubation was last for 40 min protected from light at room temperature. After that, five volumes of – 20 ℃ pre-chilled acetone was added to precipitate the proteins overnight at – 20 ℃. The precipitates were washed by 1 mL pre-chilled 90% acetone aqueous solution for twice and then re-dissolved in 100 μL 100 mM TEAB. Sequence grade modified trypsin (Promega, Madison, WI) was added at the ratio of 1:50 (enzyme: protein, weight: weight) to digest the proteins at 37 ℃ overnight. The peptide mixture was desalted by C18 ZipTip, quantified by Pierce™ Quantitative Colorimetric Peptide Assay and then lyophilized by SpeedVac.

#### Establishment of spectral database

For library generation by data dependent acquisition (DDA), 10 μg from each 30 samples were pooled as a mixture and fractionated by high pH separation with 10 fractions. The peptide mixture was re-dissolved in 50 μL of buffer A (buffer A: 20 mM ammonium formate in water, pH 10.0, adjusted with ammonium hydroxide), and then fractionated by high pH separation using Ultimate 3000 system (ThermoFisher scientific, MA, USA) connected to a reverse phase column (XBridge C18 column, 4.6 × 250 mm, 5 μm, (Waters Corporation, MA, USA). High pH separation was performed using a linear gradient, starting from 5% B to 45% B in 40 min (B: 20 mM ammonium formate in 80% ACN, pH 10.0, adjusted with ammonium hydroxide). The column was re-equilibrated at the initial condition for 15 min. The column flow rate was maintained at 1 mL/min and the column temperature was maintained at 30 ℃. Ten fractions were collected; each fraction was dried in a vacuum concentrator for the next step.

The peptides were re-dissolved in solvent A (A: 0.1% formic acid in water) and analyzed by on-line nanospray LC–MS/MS on an Orbitrap Lumos coupled to Easy-nLC 1200 system (Thermo Fisher Scientific, MA, USA). 3 μL peptide sample was loaded on an analytical column (Acclaim PepMap C18, 75 μm × 25 cm) and separated with 120-min gradient, from 9 to 32% B (B: 0.1% formic acid in ACN). The column flow rate was maintained at 600 nL/min. The electrospray voltage of 2 kV was used.

The mass spectrometer was run under data dependent acquisition mode, and automatically switched between MS and MS/MS mode. The parameters were: (1) MS: scan range (m/z)  = 350–1500; resolution  = 60,000; AGC target  = 4e^5^; maximum injection time  = 50 ms; dynamic exclusion  = 30 s; (2) HCD-MS/MS: resolution  = 30,000; AGC target  = 5e^4^; maximum injection time  = 54 ms; collision energy  = 32.

#### DIA analysis

All 30 samples were processed by DIA individually to access the proteome differences and samples acquisition by random order. The peptides were re-dissolved in 30 μL solvent A (A: 0.1% formic acid in water), 9 μL was taken out, and 1 μL 10 ×  iRT kit (Ki3002, Biognosys AG, Switzerland) was added to all of the samples to calibrate the retention time of extracted peptide peaks. 4 μL peptide sample was loaded onto the analytical column with the gradient from 6 to 32% B and other conditions were the same as the method described above.

The mass spectrometer in DIA analysis was run under DIA mode, and automatically switched between MS and MS/MS mode. The parameters were: (1) MS: scan range (m/z)  = 350–1350; resolution  = 1,20,000; AGC target  = 4e^5^; maximum injection time  = 50 ms; (2) HCD-MS/MS: resolution  = 30,000; AGC target  = 3e^5^; collision energy  = 32; (3) DIA was performed with variable isolation window, and each window overlapped 1 m/z, and the window number was 60.

#### Validation by ELISA analysis

The plasma levels of ALDOC were measured using an ELISA kit in accordance with the manufacturer’s protocols (SED320Hu, Cloud Clone, China). The optical density values were detected using a microplate reader (Tecan, Switzerland) at 450 nm, and the concentrations were automatically calculated according to the standard curve.

### Nontargeted lipidomics

#### Sample preparation

For plasma lipidomics analysis, a modified Folch method was applied. Briefly, 80 μL of plasma was spiked with 3.2 mL of extraction solvents (chloroform/methanol 2:1, v/v) and 10 μL of internal standards mixture, containing LPC (13:0) (80 μg/mL) and d5-TG-(17:0/17:1/17:0) (8 μg/mL), and then the pooled mixture was vortexed for 3 min. The mixture was subsequently kept in an ice water bath and sonicated for 3 min. 640 μL of cold water was further added to induce phase separation followed by an incubation at 4 ℃ for 10 min. After centrifugation for 10 min with 12,000 rpm at 4 ℃, the lower chloroform layer was collected, dried under a stream of nitrogen. Then the residue was reconstituted in 80 μl of acetonitrile/isopropanol (1:1, v/v), vortexed for 3 min, sonicated in an ice water bath for 3 min, and centrifuged at 16,000 rpm for 10 min for UHPLC-Q-TOF/MS analysis.

#### LC-QTOF-MS analysis

The lipid extracts were analyzed on an Agilent 1260 Infinity HPLC system coupled with an AB SCIEX TripleTOF™ 5600 triple-time-of-flight hybrid mass spectrometer system equipped with a DuoSprayTM ion source. A XSelect CSH C18 (100 × 2.1 mm, 2.6 μm) (Waters, Milford, MA) column maintained at 50 ℃ at a flow rate of 0.35 mL/min was used for chromatographic separation. The mobile phase system consisted of an acetonitrile–water mixture (60:40, v/v; solvent A) and an isopropanol-acetonitrile–water mixture (88:10:2, v/v; solvent B), both containing 10 mM ammonium formate and 0.1% formic acid. A gradient elution was carried out in positive ion mode as 10–15% B from 0 to 1 min, 15–64% B from 1 to 4 min, 64–78% B from 4 to 14 min, 100–100% B from 14.01 min to 19 min, and 10% B from 19.01 min to 26 min to equilibrate. A short analysis time was utilized to obtain a rapid method since there were less analytes in negative ion mode. The gradient in negative ion mode was programmed as follows: 10–68% B from 0 to 2 min, 68–78% B from 2 to 8 min, 78–100% B from 8 to 9 min, 100–100% B from 9 to 12 min, and 10% B from 12.01 min to 19 min.

The MS and MS/MS detection of lipids was operated in both positive and negative ion modes. Optimized parameters are listed in Additional file 1: Table S2. Nitrogen was used as a nebulizer and auxiliary gas. Scan data was collected in the mass range of 50–1600 da. Continuous recalibration solution was inserted into each five injections to correct the small mass drift in the acquisition process. The 1.7 version Analyst software (AB Sciex, USA) was utilized for the operation.

#### Data processing and bioinformatics analysis

For proteomic analysis, raw data of DDA were processed and analyzed by Spectronaut 13 (Biognosys AG, Switzerland) with default settings to generate an initial target list. Assuming trypsin as the digestion enzyme. Carbamidomethyl (C) was specified as the fixed modification. Oxidation (M) was specified as the variable modifications. Raw data of DIA were processed and analyzed by Spectronaut 13 with default settings, retention time prediction type was set to dynamic iRT. Data extraction was determined by Spectronaut 13 based on the extensive mass calibration. Spectronaut 13 was utilized to determine the ideal extraction window dynamically depending on iRT calibration and gradient stability. Qvalue (FDR) cutoff on precursor and protein level was applied 1%. Decoy generation was set to mutated which similar to scrambled but will only apply a random number of AA position swamps (min  = 2, max  = length/2). All selected precursors passing the filters were used for quantification. MS2 interference removed all interfering fragment ions except for the 3 least interfering ones. The average top 3 filtered peptides which passed the 1% Qvalue cutoff were used to calculate the major group quantities. Different expressed proteins were filtered if their FDR value  < 0.05 and fold change  > 1.5. Functions of these proteins were illustrated based on Gene ontology (GO) annotation and KEGG pathway analysis.

For lipidomic analysis, the raw LC-QTOF-MS data were firstly processed by MarkerView v1.3.1 software (AB SCIEX, USA), after peak picking, alignment, filtering, as well as data correction and normalization by internal standards, “80% rule” was implemented to retain high frequency variables, and missing values that remained were then imputed with half-the-minimum values. The generated data matrix was imported into SIMCA-P 14.1 (Umetrics, Umea, Sweden) for multivariate analyses of unsupervised principal component analysis (PCA) and supervised orthogonal partial least squares discriminant analysis (OPLS-DA), a 200-repeated permutation test was performed for OPLS-DA model to avoid overfitting and verify the reliability. Lipids with VIP scores  > 1 and p value  < 0.05 were considered prominent contributed for the model. For lipid identification, the accurate m/z, MS/MS fragment pattern and retention time were matched with public databases, including LIPID MAPS (http://www.lipidmaps.org), HMDB (http://www.hmdb.ca/) and METLIN (http://metlin.scripps.edu/). Metabolic pathway analysis, cluster analysis and heatmap representation of significantly changed lipids were conducted using MetaboAnalyst (http://www.metaboanalyst.ca/).

Pearson correlation coefficients among differently expressed proteins and lipid metabolites were calculated using SPSS and visualized as heatmaps by Heml software [9].

## Results

### Dysregulated proteins in lung cancer patients and proteome differences between syndromes

To explore the effect of QDYD and QDLS NSCLC on human plasma proteomics, DIA technology was applied to collect protein data from patients and healthy subjects. For quality control of the proteomics analysis, the results suggested that the mass spectrometry performance was stable, the accuracy of mass spectrometry was perfect, and that the data was globally normalized to the median peptide signal, leading to reliable proteomics data. (Additional file 1: Figure S1).

The initial target list from LC–MS in DDA mode contained 17,466 precursors, 11,007 peptides, 1639 proteins and 1533 protein groups. In DIA analysis, a total of 121 proteins were identified as differential proteins among groups. Thereinto, 115 proteins were altered in NSCLC patients. When compared with the healthy group, 90 (66 upregulated, 24 downregulated) and 71 (60 upregulated, 11 downregulated) proteins were changed in the QDLS and QDYD groups, respectively. Interestingly, 26 proteins could clearly distinguish the two syndrome groups, of which 12 proteins were higher in patients with QDLS while 14 proteins were the opposite. Among these differential proteins, 20 proteins overlapped with the differential proteins between NSCLC patients and healthy subjects (Additional file 1: Figure S2A). A complete list of identified proteins is provided in Additional file 2: Table S3.

**Fig. 2:**
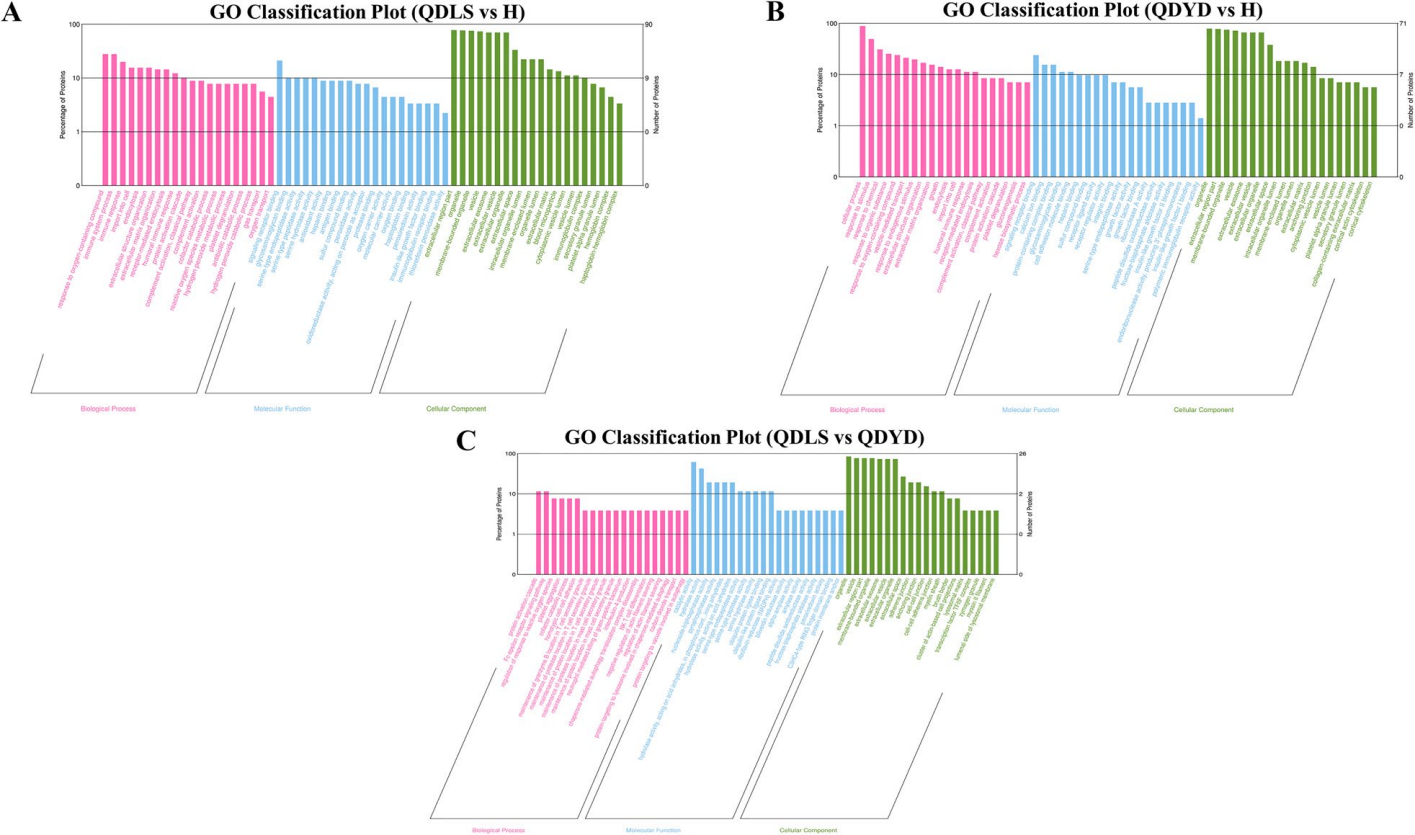
The histograms in gene ontology (GO) analysis of the differentially expressed proteins

Further GO annotation analysis revealed that dysregulated proteins in lung cancer patients were involved in reactive oxygen species metabolic process, extracellular matrix organization, growth, lipid response, and phospholipid homeostasis. Proteins significantly dysregulated between the two syndromes were mainly enriched in the biological process of cell adhesion, regulation of response to reactive oxygen species, glycolysis, and phospholipase C activity regulation (Fig. 2) (Additional file 3: Table S4). In addition, differentially expressed proteins in patients were enriched in various biological pathways in KEGG categories, such as, PI3K Akt signaling pathway, ECM-receptor interaction, glycolysis, and phospholipase D signaling pathway. However, fructose and mannose metabolism and so on were the main related KEGG pathways involved in the differential proteins between QDLS and QDYD syndromes (Additional file 3: Table S4).

Many pathways appeared more or less active for different cancer subtypes. To gain insight into pathways that would distinguish between proteome-based cancer subtypes, we explored them further below. Overall, among these biological processes and pathways, lipid response, phospholipase C activity regulation, phospholipid homeostasis, and phospholipase D signaling pathway suggested that the identified proteins in these processes may fine-tune lipids in lung cancer. Moreover, lipid metabolism was directly involved in the tumor process of lung cancer and could reflect the holistic disease state of patients with biomarkers, providing space for further exploration of lipid metabolism in NSCLC patients with different syndromes. Meanwhile, these proteins could be the key for individualized diagnosis of NSCLC.

### Characteristic lipids in lung cancer patients and lipid differences between syndromes

To validate the proteomics results and further explore the effect of the two types of NSCLC on human plasma lipid metabolism, LC-Q-TOF/MS technology was applied to collect both positive and negative ion scan data from patients and healthy subjects, and the results revealed numerous lipid changes among the different lung cancer syndromes and healthy groups.

For LC-Q-TOF/MS method validation, quality control (QC) pooled samples, prepared by mixing equal aliquots of each sample, served to assess the repeatability, stability and precision of the developed lipidomic analytical method [10]. These results showed that the developed method had good stability, repeatability and reliability for lipid analysis of human plasma (Additional file 1: Tables S5, S6).

**Fig. 3:**
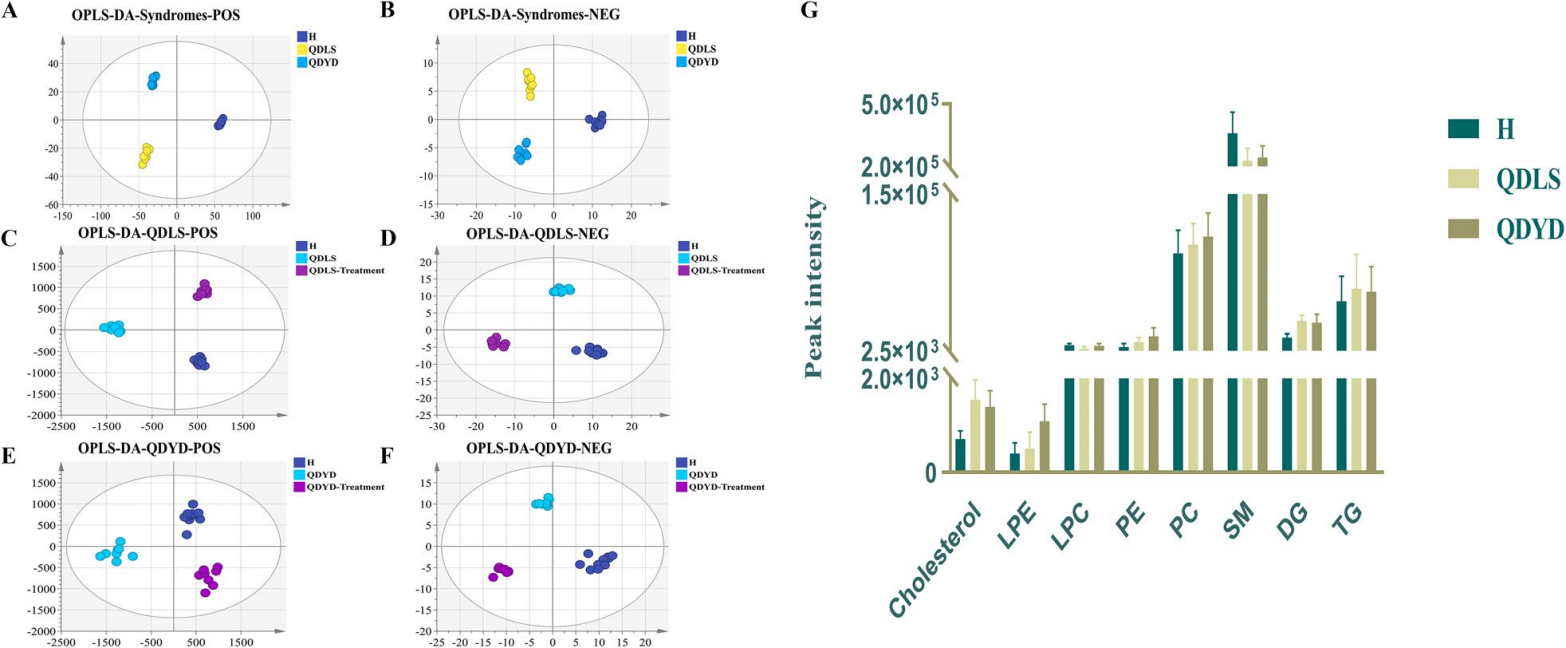
OPLS-DA score plots of lipidomic analysis in plasma from human. **A**, **B** are score plots of healthy, QDLS and QDYD patients in positive mode (R^2^  = 0.99, Q^2^  = 0.824) and negative mode (R^2^  = 0.976, Q^2^  = 0.787); **C**, **D** are score plots of healthy, QDLS patients before and after treatment in positive mode (R^2^  = 0.99, Q^2^  = 0.916) and negative mode (R^2^  = 0.985, Q^2^  = 0.867); **E**, **F** are score plots of healthy, QDYD patients before and after treatment in positive mode (R^2^  = 0.948, Q^2^  = 0.844) and negative mode (R^2^  = 0.976, Q^2^  = 0.875); **G** Total content of individual lipid class in the healthy and patient groups. *LPE* lysophosphatidylethanolamine; *LPC* lysophosphatidylcholine; *PE* phosphatidylethanolamine; *PC* phosphatidylcholine; *SM* sphingomyelin; *DG* diacylglycerol; *TG* triacylglycerol

In the score plots of the PCA models, QC samples were clustered, and both patient groups showed a clear separation trend from the healthy group. The two syndrome groups were also segregated into distinct clusters (Additional file 1: Fig. S3A, B). This result suggested that the metabolism of lung cancer patients was disordered, and the pathogenesis and clinical manifestations of the two syndromes may also be different. Moreover, the results from patients given Kangai injection were different from those of lung cancer patients with different syndromes (Additional file 1: Fig. S3C–F). Therefore, these disordered lipid metabolites could be adjusted by Kangai injection, which could be supporting evidence for the clinical value of these metabolites. Furthermore, a supervised analysis OPLS-DA was performed to extract the differential molecules (Fig. 3A–F). The results of permutation tests are shown in Additional file 1: Fig. S4, suggesting no overfitting phenomenon.

According to the principle that VIP  > 1 and p  < 0.05 were considered differential, a total of 73 lipids with significant changes were identified. 66 lipids were altered in lung cancer patients when compared with healthy group. 51 (38 upregulated, 13 downregulated) and 49 (40 upregulated, 9 downregulated) lipids were changed in the QDLS and QDYD groups, respectively (Additional file 1: Figure S2B). Of these lipids, phosphatidylcholine (PC), phosphatidylethanolamine (PE), lysophosphatidylethanolamine (LPE), diacylglycerol (DG), triacylglycerol (TG), cholesterol and ceramide (Cer) levels were elevated in the plasma samples of NSCLC patients, while some sphingomyelin (SM), and lysophosphatidylcholine (LPC) levels were down-regulated. Notably, when comparing the lipidome data in the QDLS group and QDYD group, 10 lipids including LPC, LPE, PC, PE, and TG, were distinctly different between patients with QDYD and QDLS, and the total content of LPCs of LPEs in the QDYD and QDLS groups was also prominently discrepant. Among these differential lipids, PC, PE, DG, TG, SM, LPC, LPE and cholesterol levels could be inversely regulated by Kangai injection with significant changes, while Cer level was also regulated without statistical significance. Thus, the rationality of selected markers was confirmed by Kangai injection. Detailed information was shown in Tables 1, 2, 3.

**Table 1:** Detailed information of potential biomarkers from QDLS compared to QDYD syndrome lung cancer patients

No.	m/z	t_(R)_	Identification	Quasi-molecular ion	Formula	VIP	FC	AUC	Trend
1	474.2971	1.6	LPC (O-14:1)	[M + Na]^+^	C_22_H_46_NO_6_P	1.96	7.44**	0.91	↑
2	518.3212	3.2	LPC (16:0)	[M + Na]^+^	C_24_H_50_NO_7_P	2.27	0.0029**	1.00	↓
3	564.3279	2.8	LPC (18:2)	[M + HCOO]^−^	C_26_H_50_NO_7_P	2.02	0.09**	0.91	↓
4	590.3218	2.7	LPC (22:6)	[M + Na]^+^	C_30_H_50_NO_7_P	1.41	0.56*	0.80	↓
5	454.2926	3.8	LPE (16:0)	[M + H]^+^	C_21_H_44_NO_7_P	1.55	0.40*	0.88	↓
6	526.2925	2.7	LPE (22:6)	[M + H]^+^	C_27_H_44_NO_7_P	1.41	0.57*	0.83	↓
7	758.5698	11.2	PC (16:0/18:2)	[M + H]^+^	C_42_H_80_NO_8_P	1.49	0.52*	0.84	↓
8	792.5924	10.7	PC (P-18:1/20:4)	[M + H]^+^	C_46_H_82_NO_7_P	1.46	0.58*	0.84	↓
9	766.5394	10.9	PE (16:0/22:5)	[M + H]^+^	C_43_H_76_NO_8_P	1.45	0.19*	0.83	↓
10	942.7532	17.4	TG (18:2/20:4/20:5)	[M + NH4]^+^	C_61_H_96_O_6_	1.51	0.10*	0.83	↓

*QDLS* for whom suffered Qi deficiency of lung-spleen; *QDYD* for whom suffered Qi deficiency and Yin deficiency; *FC* fold change, the ion intensity ratio of potential biomarkers from QDLS/QDYD by using the developed UHPLC-Q-TOF/MS method

*p  < 0.05 and **p  < 0.01, QDLS vs. QDYD group

**Table 2:** Detailed information of potential biomarkers from QDLS syndrome lung cancer patients compared to healthy volunteers

No.	m/z	t_(R)_	Identification	Quasi-molecular ion	Formula	VIP	FC1	FC2	AUC	Trend
1	468.3080	2.1	LPC (14:0)	[M + H]^+^	C_22_H_46_NO_7_P	1.12	0.46**	1.24	0.86	↓
2	564.3279	2.8	LPC (18:2)	[M + HCOO]^−^	C_26_H_50_NO_7_P	1.41	0.42**	1.24	0.89	↓
3	720.5894	11.6	PC (O-16:0/16:0)	[M + H]^+^	C_40_H_82_NO_7_P	1.72	1.65*	1.09	0.83	↑
4	746.5686	10.7	PC (16:0/17:1)	[M + H]^+^	C_41_H_80_NO_8_P	1.46	2.59*	0.9	0.90	↑
5	768.5885	10.8	PC (O-16:0/20:4)	[M + H]^+^	C_44_H_82_NO_7_P	3.55	0.74*	1.2	0.76	↑
6	774.6002	11.5	PC (17:0/18:1)	[M + H]^+^	C_43_H_84_NO_8_P	2.06	1.88*	0.89	0.83	↑
7	782.5670	11	PC (18:1/16:0)	[M + Na]^+^	C_42_H_82_NO_8_P	1.54	1.70**	0.76	0.88	↑
8	794.5983	9.9	PC (P-18:0/20:4)	[M + H]^+^	C_46_H_84_NO_7_P	1.46	9.72**	0.13^##^	0.94	↑
9	808.5820	11.4	PC (18:2/18:0)	[M + Na]^+^	C_44_H_84_NO_8_P	1.84	2.01**	0.60^##^	1.00	↑
10	810.5980	12	PC (18:1/18:0)	[M + Na]^+^	C_44_H_86_NO_8_P	1.62	1.90**	0.67^##^	0.96	↑
11	822.6365	11.7	PC(P-20:0/20:4)	[M + H]^+^	C_48_H_88_NO_7_P	1.26	1.51*	0.89	0.79	↑
12	830.5652	10.3	PC (18:1/20:4)	[M + Na]^+^	C_46_H_82_NO_8_P	1.02	3.88**	0.24^##^	1.00	↑
13	832.5827	11.2	PC (20:4/18:0)	[M + Na]^+^	C_46_H_84_NO_8_P	1.43	3.06**	0.45^##^	1.00	↑
14	834.5987	11.6	PC (20:3/18:0)	[M + Na]^+^	C_46_H_86_NO_8_P	1.22	1.60**	0.55^##^	0.98	↑
15	716.5232	10.6	PE (16:0/18:2)	[M + H]^+^	C_39_H_74_NO_8_P	1.43	2.43*	0.79	0.80	↑
16	740.5218	10.5	PE (16:0/20:4)	[M + H]^+^	C_41_H_74_NO_8_P	1.15	2.05*	0.57^#^	0.89	↑
17	703.5732	10.1	SM (d18:1/16:0)	[M + H]^+^	C_39_H_79_N_2_O_6_P	8.01	0.74**	1.60^##^	0.91	↓
18	785.6531	12.3	SM (d18:2/22:0)	[M + H]^+^	C_45_H_89_N_2_O_6_P	4.8	0.59**	1.14	0.85	↓
19	787.6683	13.2	SM (d18:1/22:0)	[M + H]^+^	C_45_H_91_N_2_O_6_P	7.28	0.50**	1.27	0.89	↓
20	801.6831	13.9	SM (d18:1/23:0)	[M + H]^+^	C_46_H_93_N_2_O_6_P	3.56	0.52*	1.08	0.81	↓
21	803.6235	9.4	SM (d38:1)	[M + HCOO]^−^	C_43_H_87_N_2_O_6_P	1.5	0.22**	0.9	0.91	↓
22	815.7001	14.5	SM (d18:1/24:0)	[M + H]^+^	C_47_H_95_N_2_O_6_P	6.84	0.44*	1.2	0.85	↓
23	817.6405	9.8	SM (d39:1)	[M + HCOO]^−^	C_44_H_89_N_2_O_6_P	1.4	0.10**	2.12	0.88	↓
24	827.6993	13.5	SM (d18:2/25:0)	[M + H]^+^	C_48_H_95_N_2_O_6_P	1.46	2.52*	0.82	0.81	↑
25	833.6502	12.2	SM (d18:2/24:1)	[M + Na]^+^	C_47_H_91_N_2_O_6_P	1.7	2.49**	0.69^##^	1.00	↑
26	843.6582	9.9	SM (d41:2)	[M + HCOO]^−^	C_46_H_91_N_2_O_6_P	1.38	0.22**	1.1	0.96	↓
27	591.4952	13.1	DG (16:0/18:3)	[M + H]^+^	C_37_H_66_O_5_	1.71	1.84**	0.59^##^	0.98	↑
28	607.5652	15.5	DG (18:0/18:0)	[M + H–H_2_O]^+^	C_39_H_76_O_5_	3.04	2.00**	0.46^##^	0.99	↑
29	619.5262	14.3	DG (16:0/18:0)	[M + Na]^+^	C_37_H_72_O_5_	2.93	2.16**	0.61^##^	1.00	↑
30	643.5260	13.3	DG (18:1/18:1)	[M + Na]^+^	C_39_H_72_O_5_	1.24	6.03**	0.56	0.95	↑
31	834.7539	18.4	TG (15:0/16:0/18:2)	[M + NH4]^+^	C_52_H_96_O_6_	1.17	2.22*	0.66	0.83	↑
32	836.7693	18.6	TG (15:0/16:0/18:1)	[M + NH4]^+^	C_52_H_98_O_6_	1.28	4.15**	0.54	0.94	↑
33	853.7252	18.5	TG (16:0/18:2/18:3)	[M + H]^+^	C_55_H_96_O_6_	1.19	2.31**	0.52^#^	0.93	↑
34	855.7433	18.3	TG (16:0/18:1/18:3)	[M + H]^+^	C_55_H_98_O_6_	1.79	2.51**	0.54^#^	0.96	↑
35	857.7583	18.5	TG (16:0/18:1/18:2)	[M + H]^+^	C_55_H_100_O_6_	1.06	3.98**	0.54^#^	1.00	↑
36	862.7854	18.6	TG (16:0/17:1/18:1)	[M + NH4]^+^	C_54_H_100_O_6_	1.93	2.98*	0.88	0.83	↑
37	864.8012	18.9	TG (16:0/17:0/18:1)	[M + NH4]^+^	C_54_H_102_O_6_	1.83	3.37*	0.82	0.79	↑
38	881.7579	18.7	TG (16:0/18:1/20:4)	[M + H]^+^	C_57_H_100_O_6_	2.31	2.38**	0.61^##^	1.00	↑
39	883.7702	19	TG (16:0/18:1/20:3)	[M + H]^+^	C_57_H_102_O_6_	1.7	4.39**	0.50^#^	0.93	↑
40	888.8010	18.6	TG (17:0/18:1/18:2)	[M + NH4]^+^	C_56_H_102_O_6_	2.04	2.75*	0.77	0.81	↑
41	892.7380	17.7	TG (14:0/18:2/22:6)	[M + NH4]^+^	C_57_H_94_O_6_	1.23	0.33*	0.68	0.19	↓
42	892.8325	19.1	TG (17:0/18:0/18:1)	[M + NH4]^+^	C_56_H_106_O_6_	1.27	14.99*	0.39	0.93	↑
43	894.7542	17.7	TG (18:2/18:2/18:3)	[M + NH4]^+^	C_57_H_96_O_6_	4.35	0.37*	0.99	0.13	↓
44	907.7727	18.8	TG (18:1/18:1/20:4)	[M + H]^+^	C_59_H_102_O_6_	1.52	2.42**	0.56^##^	0.96	↑
45	909.7853	19	TG (18:0/18:1/18:1)	[M + Na]^+^	C_57_H_106_O_6_	1.39	3.89**	0.62	0.95	↑
46	918.7540	17.6	TG (18:2/18:2/20:5)	[M + NH4]^+^	C_59_H_96_O_6_	1.54	0.37*	0.88	0.16	↓
47	928.8294	18.8	TG (18:0/18:1/20:3)	[M + NH4]^+^	C_59_H_106_O_6_	1.61	1.75*	0.73	0.78	↑
48	932.8636	19.2	TG (16:0/18:1/22:1)	[M + NH4]^+^	C_59_H_110_O_6_	1.09	2.12*	0.52	0.81	↑
49	952.8320	18.7	TG (18:1/18:1/22:4)	[M + NH4]^+^	C_61_H_106_O_6_	1.12	3.54**	0.46	0.90	↑
50	630.6178	14.6	Cer (d18:1/24:1)	[M + H–H_2_O]^+^	C_42_H_81_NO_3_	1.03	2.29*	0.78	0.74	↑
51	369.3521	10.3	Cholesterol	[M + H–H_2_O]^+^	C_27_H_46_O	1.21	2.18*	0.45^##^	0.99	↑

*QDLS* for whom suffered Qi deficiency of lung-spleen; *FC1* the ion intensity ratio of potential biomarkers from QDLS/healthy volunteers by using the developed UHPLC-Q-TOF/MS method; *FC2* the ion intensity ratio of potential biomarkers from QDLS-Treatment/QDLS by using the developed UHPLC-Q-TOF/MS method, QDLS-Treatment for whom suffered Qi deficiency of lung-spleen and treated by Kangai Injection

^*^p  < 0.05 and  **p  < 0.01, QDLS vs. H group

^#^*p*  < 0.05 and ^##^*p*  < 0.01,  QDLS-Treatment vs. QDLS group

**Table 3:** Detailed information of potential biomarkers from QDYD syndrome lung cancer patients compared to healthy volunteers

No.	m/z	t_(R)_	Identification	Quasi-molecular ion	Formula	VIP	FC1	FC2	AUC	Trend
1	494.3244	2.5	LPC (16:1)	[M + H]^+^	C_24_H_48_NO_7_P	1.47	0.64*	1.64^#^	0.81	↓
2	454.2926	3.8	LPE (16:0)	[M + H]^+^	C_21_H_44_NO_7_P	1.21	4.31**	0.44^##^	0.98	↑
3	730.5377	9.6	PC (14:0/18:2)	[M + H]^+^	C_40_H_76_NO_8_P	2.72	0.47**	1.21	0.94	↓
4	746.5686	10.6	PC (16:0/17:1)	[M + H]^+^	C_41_H_80_NO_8_P	1.13	1.64**	0.78	0.88	↑
5	780.5517	11.2	PC (16:0/18:2)	[M + Na]^+^	C_42_H_80_NO_8_P	1.65	2.45**	0.60^##^	1.00	↑
6	774.6002	11.5	PC (17:0/18:1)	[M + H]^+^	C_43_H_84_NO_8_P	1.56	1.54*	0.8	0.84	↑
7	782.5670	11	PC (18:1/16:0)	[M + Na]^+^	C_42_H_82_NO_8_P	1.61	1.91**	0.64^##^	0.98	↑
8	794.5983	9.9	PC (P-18:0/20:4)	[M + H]^+^	C_46_H_84_NO_7_P	1.11	38.44**	0.17^#^	0.91	↑
9	806.5673	10.6	PC (16:0/20:3)	[M + Na]^+^	C_44_H_82_NO_8_P	1.83	1.89**	0.62^##^	1.00	↑
10	808.5820	11.4	PC (18:2/18:0)	[M + Na]^+^	C_44_H_84_NO_8_P	1.86	1.93**	0.64^##^	1.00	↑
11	810.5980	12.1	PC (18:1/18:0)	[M + Na]^+^	C_44_H_86_NO_8_P	1.72	1.94**	0.62^##^	0.98	↑
12	828.5514	10	PC (16:0/22:6)	[M + Na]^+^	C_46_H_80_NO_8_P	1.51	1.73**	0.66^##^	1.00	↑
13	830.5652	10.3	PC (18:1/20:4)	[M + Na]^+^	C_46_H_82_NO_8_P	1.41	3.55**	0.29^##^	1.00	↑
14	832.5827	11.2	PC (20:4/18:0)	[M + Na]^+^	C_46_H_84_NO_8_P	1.19	2.56**	0.56^#^	0.94	↑
15	834.5987	11.5	PC (20:3/18:0)	[M + Na]^+^	C_46_H_86_NO_8_P	1.23	1.62**	0.62^##^	0.94	↑
16	716.5232	10.6	PE (16:0/18:2)	[M + H]^+^	C_39_H_74_NO_8_P	1.79	2.80**	0.66	0.93	↑
17	740.5218	10.5	PE (16:0/20:4)	[M + H]^+^	C_41_H_74_NO_8_P	1.75	3.15**	0.50^#^	0.95	↑
18	764.5227	10.3	PE (16:0/22:6)	[M + H]^+^	C_43_H_74_NO_8_P	1.41	3.32**	0.56	0.95	↑
19	768.5534	11.4	PE (18:0/20:4)	[M + H]^+^	C_43_H_78_NO_8_P	2.87	2.31**	0.71	0.84	↑
20	703.5732	10.1	SM (d18:1/16:0)	[M + H]^+^	C_39_H_79_N_2_O_6_P	9.05	0.72**	1.59^##^	0.94	↓
21	733.6212	11.4	SM (d36:0)	[M + H]^+^	C_41_H_85_N_2_O_6_P	1.16	2.03*	0.7	0.74	↑
22	787.6683	13.2	SM (d18:1/22:0)	[M + H]^+^	C_45_H_91_N_2_O_6_P	6.45	0.64*	1.24	0.76	↓
23	801.6831	13.9	SM (d18:1/23:0)	[M + H]^+^	C_46_H_93_N_2_O_6_P	3.51	0.58*	1.15	0.73	↓
24	815.7001	14.4	SM (d18:1/24:0)	[M + H]^+^	C_47_H_95_N_2_O_6_P	6.42	0.55*	1.18	0.73	↓
25	817.6405	9.8	SM (d39:1)	[M + HCOO]^−^	C_44_H_89_N_2_O_6_P	1.42	0.10**	3.05	0.88	↓
26	829.6400	9.5	SM (d40:2)	[M + HCOO]^−^	C_45_H_89_N_2_O_6_P	1.39	0.36**	0.48	0.90	↓
27	833.6502	12.2	SM (d18:2/24:1)	[M + Na]^+^	C_47_H_91_N_2_O_6_P	1.61	2.30**	0.65^##^	1.00	↑
28	843.6582	9.9	SM (d41:2)	[M + HCOO]^−^	C_46_H_91_N_2_O_6_P	1.33	0.38**	1.35	0.90	↓
29	591.4952	13.1	DG (16:0/18:3)	[M + H]^+^	C_37_H_66_O_5_	1.62	1.72**	0.58^#^	0.93	↑
30	607.5652	15.6	DG (18:0/18:0)	[M + H–H_2_O]^+^	C_39_H_76_O_5_	2.96	1.83**	0.48^##^	0.98	↑
31	619.5262	14.3	DG (16:0/18:0)	[M + Na]^+^	C_37_H_72_O_5_	2.62	1.93**	0.63^##^	0.91	↑
32	641.5107	12.4	DG (18:1/18:2)	[M + Na]^+^	C_39_H_70_O_5_	1.63	1.89**	0.7	0.89	↑
33	643.5260	13.3	DG (18:1/18:1)	[M + Na]^+^	C_39_H_72_O_5_	1.28	6.41**	0.59	0.85	↑
34	836.7693	18.6	TG (15:0/16:0/18:1)	[M + NH4]^+^	C_52_H_98_O_6_	1.06	2.95**	0.67	0.93	↑
35	855.7433	18.3	TG (16:0/18:1/18:3)	[M + H]^+^	C_55_H_98_O_6_	1.81	2.55**	0.44^##^	0.99	↑
36	862.7854	18.6	TG (16:0/17:1/18:1)	[M + NH4]^+^	C_54_H_100_O_6_	2.03	2.96**	0.67	0.96	↑
37	864.8012	18.9	TG (16:0/17:0/18:1)	[M + NH4]^+^	C_54_H_102_O_6_	1.6	2.68**	0.75	0.96	↑
38	881.7579	18.7	TG (16:0/18:1/20:4)	[M + H]^+^	C_57_H_100_O_6_	2.53	2.52**	0.59^##^	1.00	↑
39	883.7702	19	TG (16:0/18:1/20:3)	[M + H]^+^	C_57_H_102_O_6_	1.99	5.56**	0.36^##^	1.00	↑
40	888.8010	18.6	TG (17:0/18:1/18:2)	[M + NH4]^+^	C_56_H_102_O_6_	2	2.21**	0.61	0.99	↑
41	890.8145	18.9	TG (17:0/18:1/18:1)	[M + NH4]^+^	C_56_H_104_O_6_	2.28	2.55**	0.78	0.96	↑
42	909.7853	19	TG (18:0/18:1/18:1)	[M + Na]^+^	C_57_H_106_O_6_	1.68	4.76**	0.53^##^	1.00	↑
43	930.8466	19	TG (18:1/18:1/20:1)	[M + NH4]^+^	C_59_H_108_O_6_	1.5	2.27**	0.69	0.91	↑
44	932.8636	19.2	TG (16:0/18:1/22:1)	[M + NH4]^+^	C_59_H_110_O_6_	1.21	2.46**	0.57	0.86	↑
45	950.8130	18.4	TG (18:1/18:1/22:5)	[M + NH4]^+^	C_61_H_104_O_6_	1.41	2.15*	0.55	0.76	↑
46	952.8320	18.6	TG (18:1/18:1/22:4)	[M + NH4]^+^	C_61_H_106_O_6_	1.28	3.22**	0.41^##^	0.93	↑
47	960.8935	19.5	TG (18:1/18:1/22:0)	[M + NH4]^+^	C_61_H_114_O_6_	1.14	4.63**	0.33^#^	0.94	↑
48	630.6178	14.6	Cer (d18:1/24:1)	[M + H–H_2_O]^+^	C_42_H_81_NO_3_	1.04	2.16**	0.69	0.93	↑
49	369.3521	10.3	Cholesterol	[M + H–H_2_O]^+^	C_27_H_46_O	1.02	1.99**	0.48^##^	1.00	↑

*QDYD* for whom suffered Qi deficiency and Yin deficiency; *FC1* the ion intensity ratio of potential biomarkers from QDYD/healthy volunteers by using the developed UHPLC-Q-TOF/MS method; *FC2* the ion intensity ratio of potential biomarkers from QDYD-Treatment/QDYD by using the developed UHPLC-Q-TOF/MS method, QDYD-Treatment for whom suffered Qi deficiency and Yin deficiency and treated by Kangai Injection

*p  < 0.05 and **p  < 0.01, QDLS vs. H group; ^#^*p*  < 0.05 and ^##^*p*  < 0.01, QDYD-Treatment vs. QDYD group

To investigate the metabolic phenogram of lung cancer as well as the comparative relationship of both syndromes, fold changes of the altered lipids in lung cancer were compared and displayed together with the potential biomarkers through comparison between syndrome groups (Table 4). Considering a more intuitive visualization, changes in the total content of each lipid class in the three groups were exhibited (Fig. 3G). The contents of DG, TG and cholesterol were increased in both syndrome groups compared with healthy subjects. Likewise, PC and PE were increased in the patient groups, while QDYD syndrome showed a slightly higher trend. LPE levels in the QDYD group were elevated. In contrast, SM and LPC were decreased in both patient groups. Notably, the LPC and LPE levels were prominently changed between the two syndromes.

**Table 4:** Altered lipids in lung cancer and potential biomarkers in QDLS and QDYD syndromes

Metabolite	QDLS/H		QDYD/H		QDLS/QDYD	
	*p*	FC	*p*	FC	*p*	FC
LPC (18:2)	0.002	↓ 0.42	–	↓ 0.55	0.005	↓ 0.77
LPE (16:0)	–	↑ 1.72	< 0.001	↑ 4.31	0.013	↓ 0.40
PC (16:0/17:1)	0.048	↑ 2.59	0.01	↑ 1.64	–	↑ 1.58
PC (16:0/18:2)	–	↑ 1.27	< 0.001	↑ 2.45	0.018	↓ 0.52
PC (17:0/18:1)	0.017	↑ 1.88	0.045	↑ 1.54	–	↑ 1.21
PC (18:1/16:0)	0.006	↑ 1.70	< 0.001	↑ 1.91	–	↓ 0.89
PC (P-18:0/20:4)	< 0.001	↑ 9.72	0.002	↑ 38.44	–	↓ 0.25
PC (18:2/18:0)	< 0.001	↑ 2.01	< 0.001	↑ 1.93	–	↑ 1.04
PC (18:1/18:0)	< 0.001	↑ 1.90	< 0.001	↑ 1.94	–	↓ 0.98
PC (18:1/20:4)	< 0.001	↑ 3.88	< 0.001	↑ 3.55	–	↑ 1.09
PC (20:4/18:0)	< 0.001	↑ 3.06	< 0.001	↑ 2.56	–	↑ 1.19
PC (20:3/18:0)	< 0.001	↑ 1.60	< 0.001	↑ 1.62	–	↓ 0.99
PE (16:0/18:2)	0.014	↑ 2.43	< 0.001	↑ 2.80	–	↓ 0.87
PE (16:0/20:4)	0.01	↑ 2.05	< 0.001	↑ 3.15	–	↓ 0.65
SM (d18:1/16:0)	0.002	↓ 0.74	< 0.001	↓ 0.72	–	↑ 1.03
SM (d18:1/22:0)	0.007	↓ 0.50	0.042	↓ 0.64	–	↓ 0.78
SM (d18:1/23:0)	0.026	↓ 0.52	0.035	↓ 0.58	–	↓ 0.90
SM (d18:1/24:0)	0.013	↓ 0.44	0.045	↓ 0.55	–	↓ 0.8
SM (d39:1)	0.001	↓ 0.09	0.001	↓ 0.10	–	↓ 0.9
SM (d18:2/24:1)	< 0.001	↑ 2.49	< 0.001	↑ 2.30	–	↑ 1.08
SM (d41:2)	< 0.001	↓ 0.22	0.006	↓ 0.38	–	↓ 0.58
DG (16:0/18:3)	< 0.001	↑ 1.84	0.002	↑ 1.72	–	↑ 1.07
DG (18:0/18:0)	< 0.001	↑ 2.00	< 0.001	↑ 1.83	–	↑ 1.09
DG (16:0/18:0)	< 0.001	↑ 2.16	< 0.001	↑ 1.93	–	↑ 1.12
DG (18:1/18:1)	< 0.001	↑ 6.03	0.002	↑ 6.41	–	↓ 0.94
TG (15:0/16:0/18:1)	0.003	↑ 4.15	0.003	↑ 2.95	–	↑ 1.41
TG (16:0/18:1/18:3)	< 0.001	↑ 2.51	< 0.001	↑ 2.55	–	↓ 0.98
TG (16:0/17:1/18:1)	0.016	↑ 2.98	0.002	↑ 2.96	–	↑ 1.01
TG (16:0/17:0/18:1)	0.029	↑ 3.37	0.003	↑ 2.68	–	↑ 1.26
TG (16:0/18:1/20:4)	< 0.001	↑ 2.38	< 0.001	↑ 2.52	–	↓ 0.94
TG (16:0/18:1/20:3)	< 0.001	↑ 4.39	< 0.001	↑ 5.56	–	↓ 0.79
TG (17:0/18:1/18:2)	0.029	↑ 2.75	0.003	↑ 2.21	–	↑ 1.24
TG (18:0/18:1/18:1)	< 0.001	↑ 3.89	< 0.001	↑ 4.76	–	↓ 0.82
TG (16:0/18:1/22:1)	0.036	↑ 2.12	0.01	↑ 2.46	–	↓ 0.86
TG (18:1/18:1/22:4)	0.003	↑ 3.54	< 0.001	↑ 3.22	–	↑ 1.09
Cer (d18:1/24:1)	0.024	↑ 2.29	< 0.001	↑ 2.16	–	↑ 1.06
Cholesterol	< 0.001	↑ 2.18	< 0.001	↑ 1.99	–	↑ 1.10
LPC (14:0)	0.004	↓ 0.46	–	–	–	–
PC (O-16:0/16:0)	0.018	↑ 1.65	–	–	–	–
PC (O-16:0/20:4)	0.042	↓ 0.74	–	–	–	–
PC(P-20:0/20:4)	0.045	↑ 1.51	–	–	–	–
SM (d18:2/22:0)	0.009	↓ 0.59	–	–	–	–
SM (d38:1)	< 0.001	↓ 0.22	–	–	–	–
SM (d18:2/25:0)	0.019	↑ 2.52	–	–	–	–
TG (15:0/16:0/18:2)	0.039	↑ 2.22	–	–	–	–
TG (16:0/18:2/18:3)	0.005	↑ 2.31	–	–	–	–
TG (16:0/18:1/18:2)	< 0.001	↑ 3.98	–	–	–	–
TG (14:0/18:2/22:6)	0.029	↓ 0.33	–	–	–	–
TG (17:0/18:0/18:1)	0.041	↑ 14.99	–	–	–	–
TG (18:2/18:2/18:3)	0.031	↓ 0.37	–	–	–	–
TG (18:1/18:1/20:4)	< 0.001	↑ 2.42	–	–	–	–
TG (18:2/18:2/20:5)	0.041	↓ 0.37	–	–	–	–
TG (18:0/18:1/20:3)	0.019	↑ 1.75	–	–	–	–
LPC (16:1)	–	–	0.024	↓ 0.64	–	–
PC (14:0/18:2)	–	–	0.003	↓ 0.47	–	–
PC (16:0/20:3)	–	–	< 0.001	↑ 1.89	–	–
PC (16:0/22:6)	–	–	< 0.001	↑ 1.73	–	–
PE (16:0/22:6)	–	–	< 0.001	↑ 3.32	–	–
PE (18:0/20:4)	–	–	0.007	↑ 2.31	–	–
SM (d36:0)	–	–	0.048	↑ 2.03	–	–
SM (d40:2)	–	–	0.003	↓ 0.36	–	–
DG (18:1/18:2)	–	–	0.009	↑ 1.89	–	–
TG (17:0/18:1/18:1)	–	–	0.002	↑ 2.55	–	–
TG (18:1/18:1/20:1)	–	–	0.004	↑ 2.27	–	–
TG (18:1/18:1/22:5)	–	–	0.011	↑ 2.15	–	–
TG (18:1/18:1/22:0)	–	–	0.002	↑ 4.63	–	–

*QDLS* for whom suffered Qi deficiency of lung-spleen syndrome of lung cancer; *QDYD* for whom suffered Qi deficiency and Yin deficiency syndrome of lung cancer; *FC* the ratio of ion intensity for potential biomarkers from patients or healthy volunteers by using the developed UHPLC-Q-TOF/MS method

**Fig. 4:**
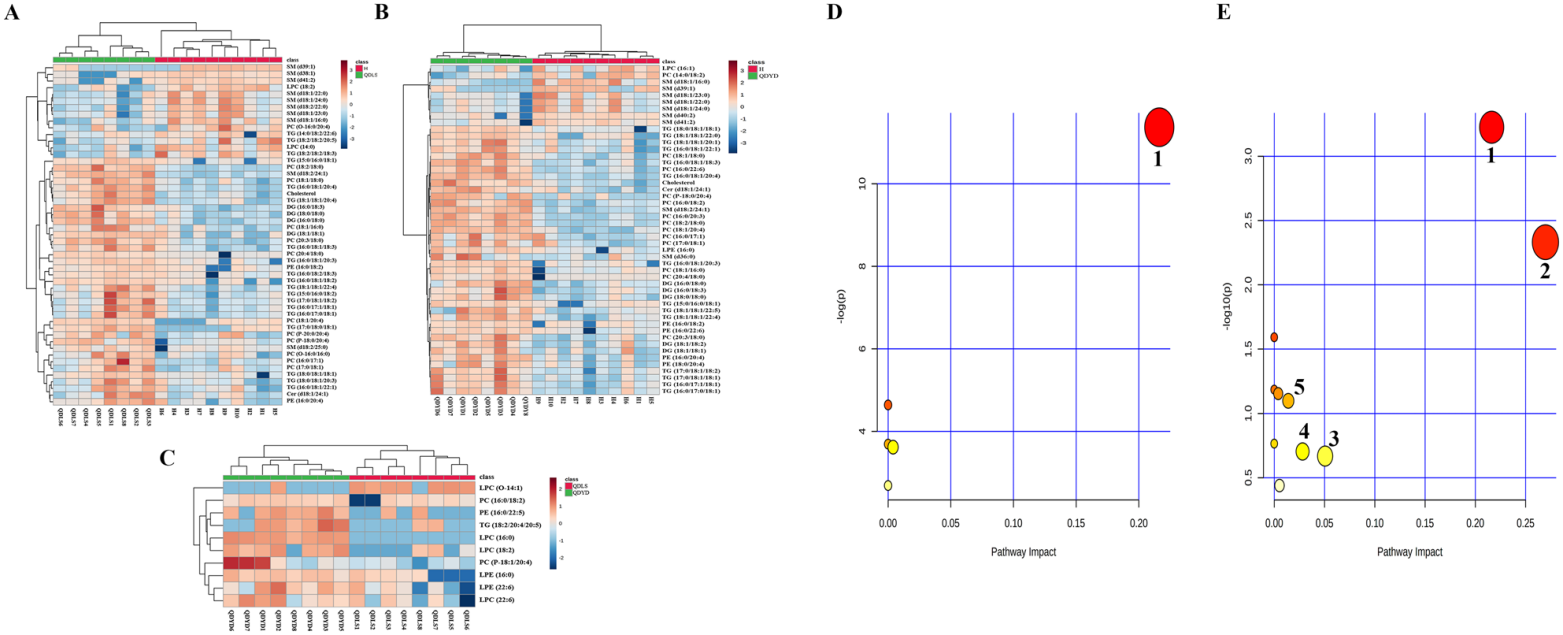
Hierarchical clustering heatmaps of potential lipid biomarkers in plasma from **A** healthy and QDLS group, **B** healthy and QDYD group, and **C** QDLS and QDYD group; **D** Pathway analysis of altered lipids between QDLS and QDYD syndromes of lung cancer; **E** Pathway analysis of altered lipids in lung cancer patients. 1, glycerophospholipid metabolism; 2, sphingolipid metabolism; 3, primary bile acid biosynthesis; 4, steroid biosynthesis; 5, glycerolipid metabolism

Heatmaps generated from the results of hierarchical clustering analysis showed the profiles of potential biomarkers between cancer and healthy subjects and between the two syndromes, which could clearly distinguish the groups in pairs (Fig. 4A–C). As a result of metabolic pathways, sphingolipid metabolism, glycerophospholipid metabolism, primary bile acid biosynthesis, steroid biosynthesis, and glycerolipid metabolism were prominently changed in lung cancer (Fig. 4E). Notably, glycerophospholipid metabolism emerged at the top of the pathway map, which was evidently altered between the two syndromes of lung cancer (Fig. 4D).

### Plasma bi-omics integration analysis

**Fig. 5:**
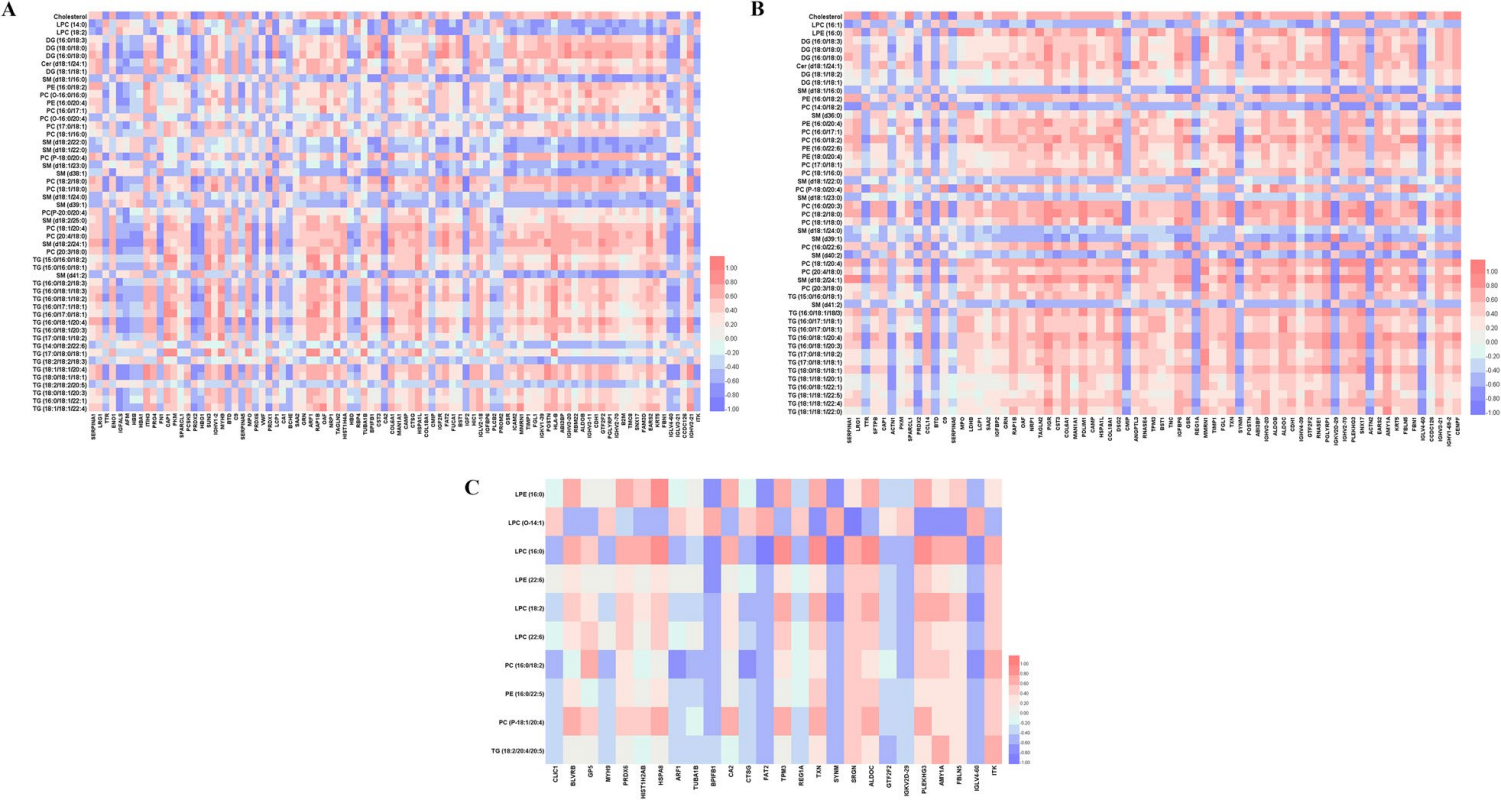
Heatmaps of correlation matrix in correlations analysis showing the relationship between proteomic and lipidomic datasets. For proteomic data, the gene names are displayed. Correlation matrix of proteomic and lipidomic results between **A** QDLS patients and healthy people, **B** QDYD patients and healthy people; **C** QDLS and QDYD patients

In the statistical correlation analysis, as shown in the correlation matrix, there were some notable correlations between lipid-based and protein-based results in the two syndromes (Fig. 5). Based on the correlation coefficient between the proteomics and lipidomics results, the relationship among altered lipids and proteins was analyzed, and the essential differences between the two syndrome groups were further explored. The results indicated that there were many proteins significantly related to lipids in specific lipid classes (Additional file 1: Table S7). For example, fructose-bisphosphate aldolase C (ALDOC) was highly correlated with LPCs, PCs and TGs, which exceeded a correlation matrix (|r|) higher than 0.8, tubulin alpha-1B chain (TUBA1B) had strong correlation with TGs. So do collagen alpha-1(VI) chain (COL6A1), desmoglein-2 (DSG2), cystatin-C (CST3), and thioredoxin (TXN). Among them, ALDOC and TUBA1B differentially expressed between QDLS syndrome and QDYD syndrome.

Additionally, we investigated biological correlations by analyzing the results above against HMDB, KEGG, MetScape, and LipidMaps databases and previous research. Proteins that were involved in lipid metabolism and correlated with certain lipids with |r| between 0.5 and 0.8 or higher than 0.8 were identified, such as ALDOC, proprotein convertase subtilisin/kexin type 9 (PCSK9), angiopoietin-related protein 3 (ANGPTL3) and peroxiredoxin-6 (PRDX6). ALDOC participates in the fructose 1,6-bisphosphate metabolic process and is involved in glycerophospholipid metabolism. PCSK9 plays a key role in the regulation of plasma cholesterol homeostasis. ANGPTL3 and PRDX6 exert regulatory effects on plasma TG levels and glycerophospholipid levels, respectively. Among them, ALDOC, PCSK9, and PRDX6 differentially expressed between QDLS syndrome and QDYD syndrome.

After the integration of statistical correlation and biological correlation, ALDOC and LPCs were screened out as differential proteins and lipids between the QDLS and QDYD syndromes of NSCLC patients, overlapped with differential substances between NSCLC patients and healthy subjects, and had consistent statistical and biological correlations. ALDOC is involved in the metabolism of glycerophospholipids, its product participates in the synthesis of PCs and PEs, and it is closely related to the metabolism of LPCs and LPEs in glycerophospholipid metabolism. In addition, ALDOC also had a statistically significant relationship with LPCs, and their AUC values were more than 0.8. Thus, they were not only statistically correlated but also jointly participate in glycerophospholipid metabolism, which evidently changed between the two syndromes of lung cancer. They contributed to syndrome differentiation of QDLS and QDYD syndromes in NSCLC. In addition, the most significant metabolic differences between the two syndromes were further discussed.

To validate the proteomic results of ALDOC, we quantified it by ELISA analysis. As shown in Additional file 1: Fig. S5, ALDOC showed statistically significant upregulation in the QDYD group compared with the QDLS group, which was consistent with the proteomic results.

## Discussion

### Proteins differently expressed in NSCLC patients

**Fig. 6:**
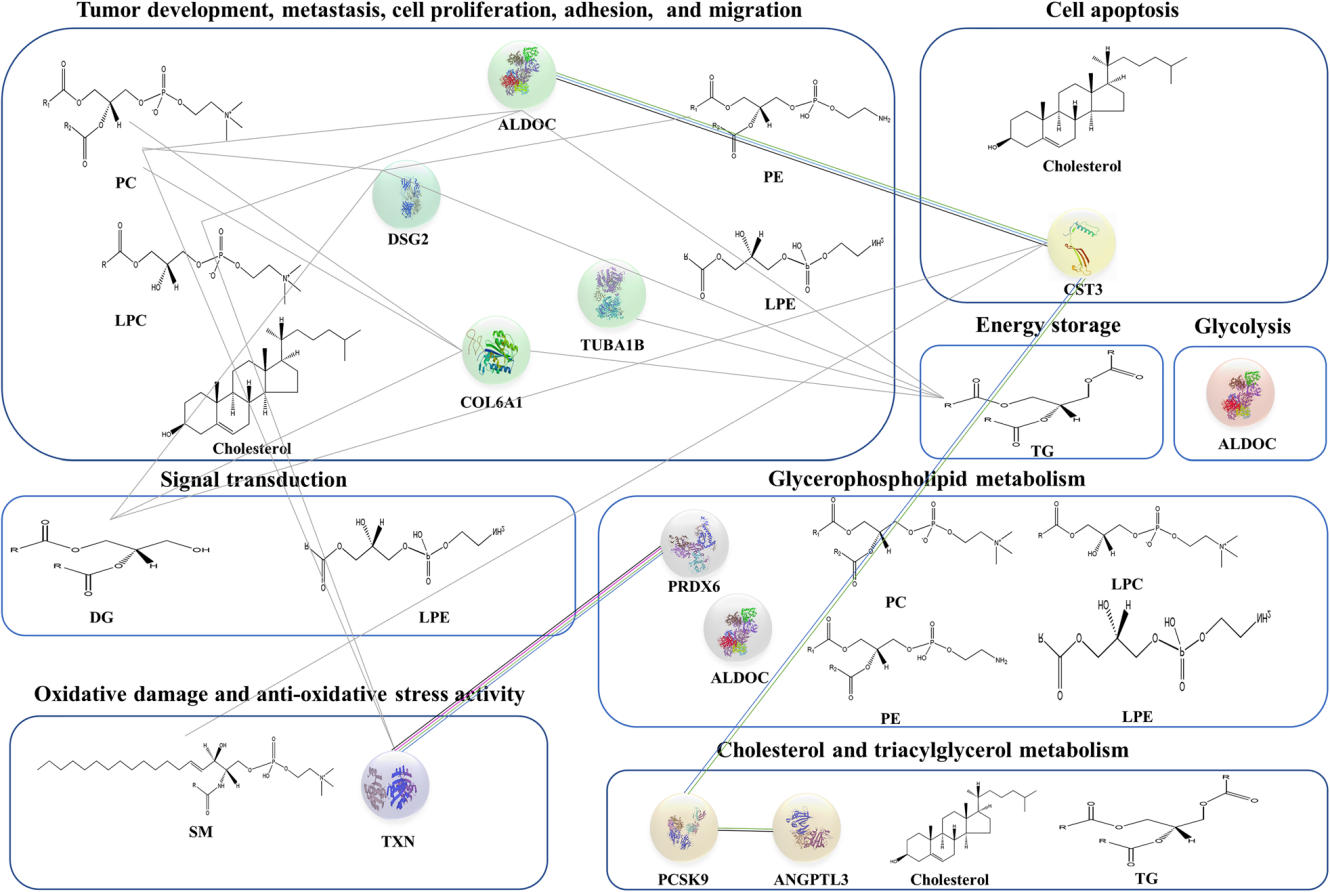
Relevance network graph depicting relationships between lipids and proteins as well as the protein–protein interactions. Based on the results in STRING, the lines between proteins represent protein–protein interactions information. The lipid structures are displayed together with protein three-dimensional structures, and the metabolic processes in which proteins and lipids are involved are labeled. The gray line represent that the protein is significantly related to the response of lipids

LC–MS-based proteomics has been proven to be a wide-ranging tool for plasma biomarker screening in recent years. All precursor ions within the selected m/z range could be fragmented in the process of DIA and analyzed in a single MS/MS scan. Thus, DIA allows for the measurement of peptides with low abundance and more accurate quantification. Multi-omics analysis is a powerful approach to jointly explore changes in the proteome and lipidome in vivo in this study. The protein–protein interactions as well as the relationship between lipids and proteins are clearly presented in Fig. 6. This strategy could be used to generate comprehensive testable hypotheses.

#### Dysregulated proteins highly correlated with the lipid response

The expression of many proteins was different between the NSCLC patient groups and the healthy group, including ALDOC, COL6A1, TUBA1B, DSG2, TXN, and CST3, and the differences were highly correlated with the lipid response, which might play crucial roles in NSCLC.

ALDOC is a member of the glycolysis enzyme family, which catalyzes the decomposition of β-D-fructose 1,6-bisphosphate into glycerone phosphate and d-glycraldehyde 3-phosphate, while its product is involved in glycerophospholipid metabolism. Meanwhile, ALDOC positively regulates the Wnt pathway, which is involved in tumor development, by blocking the GSK-3β-axin interaction and targeting axin to a Dvl-induced signalosome [11]. In addition, the glycolysis pathway in which ALDOC participates in could affect the energy metabolism of patients, and the differential expression of ALDOC may lead to different energy metabolism in patients with two syndromes, which was consistent with the influence of yin deficiency on energy metabolism [12]. In our study, ALDOC was highly correlated with LPCs and PCs in the lipidomics results, and affected the lipid metabolism of lung cancer, while the effects on QDLS and QDYD syndromes were discrepant. The role of ALDOC in the statistical correlation analysis with LPCs and PCs was consistent with that in lipid metabolism, which could contribute to syndrome differentiation as a key differential protein. Consequently, the discrepancies between QDLS and QDYD seemed to be related to the metabolic differences in glycerophospholipid metabolism and the glycolysis pathway involved in ALDOC.

COL6A1 is widely present in the extracellular matrix (ECM) and mediates the formation of microfibril networks. The ECM is emerging as an important component of the tumor microenvironment, providing structural support and regulating the activities of growth factors and cytokines. COL6A1 is reportedly a crucial regulator of lung cancer invasion and metastasis [13]. Its correlation with metastasis may be realized by changing the characteristics of ECM, promoting cell adhesion to ECM and supporting cell movement. The level of it was elevated in NSCLC patients with QDLS syndrome. As a subtype of α-tubulin, TUBA1B participates in the formation of microtubules and is generally involved in cell proliferation, adhesion, movement and division. Both G2/M cell cycle arrest and abnormal mitotic spindle formation, and subsequent apoptosis signal triggering could be caused by microtubule destruction [14]. Thus, the overexpression of TUBA1B in QDLS syndrome might be involved in the proliferation of cancer cells. DSG2, a protein of the cadherin superfamily, participates in cell adhesion and has been demonstrated to be overexpressed in NSCLC, which was consistent with the results in both syndrome groups [15]. TXN, a small molecule selenium-containing protein with a molecular weight of approximately 12 kDa, forms the thioredoxin system together with nicotinamide adenine dinucleotide phosphate-oxidase (NADPH) and thioredoxin reductase (TRXR), which is one of the two redox regulatory systems supporting tumor growth. TXN is primarily responsible for defense against the oxidative stress burden caused by elevated reactive oxygen species (ROS) in lung cancer [16]. CST3 is a member of the cysteine protease inhibitor family, which mainly exists in extracellular fluid. The levels of cystatin C in lung cancer groups were elevated, consistent with the previous report [17]. “Qi” here was vital qi, and the interplay with evil qi determines the development of cancer. The deficiency of vital qi increases the ability of evil qi pathogenic factors to do harm and can aggravate the illness. The incidence and severity of qi deficiency in advanced cancer is higher than that in early stages [3]. Therefore, qi deficiency might be related to the progression of lung cancer by the metabolic pathways involving the above proteins.

#### Differential proteins involved in lipid metabolism

**Fig. 7:**
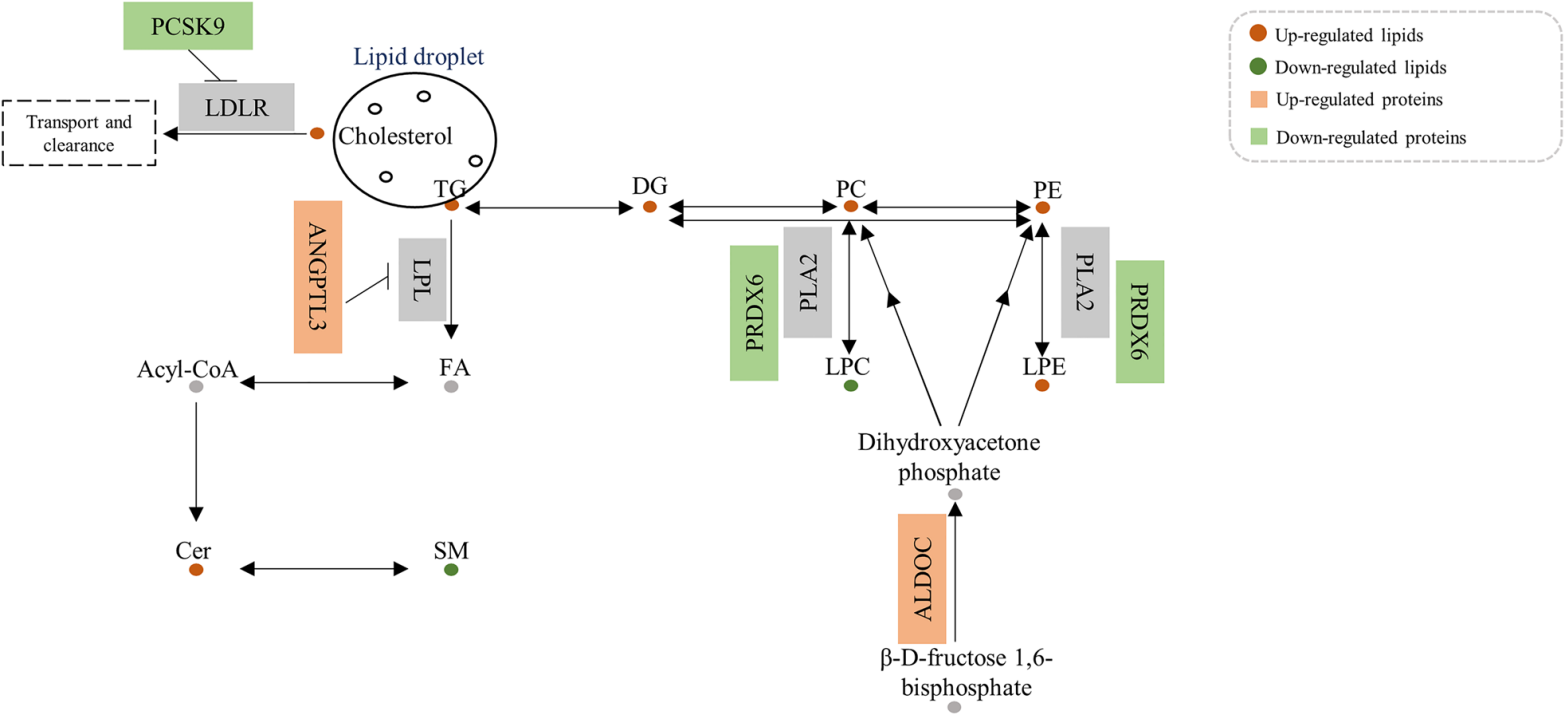
The pathway map of identified lipid and protein biomarkers involved in lipid metabolism

In addition, some differential proteins have been found to be involved in lipid metabolism based on the HMDB, KEGG, MetScape and LipidMaps databases and literature reports. These proteins, including ALDOC, PCSK9, ANGPTL3, and PRDX6, were correlated with certain lipids (Fig. 7). PCSK9 is emerging as a key regulator of plasma cholesterol homeostasis. PCSK9 in the circulation can bind to low-density lipoprotein receptor (LDLR), the receptor for LDL, which participates in cholesterol transport and clearance from the blood. PCSK9 promotes LDLR degradation and prevents its recirculation, leading to hypercholesteremia, and associated with a variety of malignant tumors [18, 19]. Consistent with the significant upregulation of cholesterol in our results, the overexpression of PCSK9 in QDLS lung cancer might become a key factor in the occurrence and development of lung cancer together with cholesterol synergistically. As one of the effective regulators of lipoprotein metabolism, ANGPTL3 inhibits the activity of lipoprotein lipase (LPL) through the N-terminal domain CCD fragment to prevent the clearance of plasma TGs [20]. The significantly elevated level of this protein in the QDYD lung cancer patients might be related to the abnormal TG levels in our results. Interestingly, ANGPTL3 had a positive correlation with certain TG species in the lipidomics results (|r| 0.5–0.8), which was consistent with the theory above. PRDX6 is a bifunctional enzyme with both peroxidase activity and phospholipase A2 activity. PRDX6 was enriched in the glycerophospholipid catabolic process by reducing the oxidized sn-2 fatty acyl group (peroxidase activity) and hydrolyzing the sn-2 ester bond (phospholipase activity) of phospholipids. LCAT could also be catalyzed by PRDX6. In summary, the membrane lipid peroxidation caused by oxidative stress could be prevented by PRDX6 to maintain the homeostasis of phospholipid metabolism. Recent studies have demonstrated that PRDX6 could activate Akt through the activation of phosphoinositide 3-kinase (PI3K) and p38 kinase, and further induce uPA (urokinase plasminogen activator) to promote the invasion of lung cancer cells [21]. In our study, PRDX6 was low expressed in QDLS lung cancer, and have a significant difference between the two syndromes, implying that PRDX6 focused on the influence of invasion ability and phospholipid metabolism homeostasis with “Yin deficiency” in the two groups of lung cancer.

##### Lipid changes in NSCLC patients

From the lipidomics screening, we observed elevated PC levels in the plasma of lung cancer patients with QDLS or QDYD syndrome. As a key component of the eukaryotic cell membrane, changes in PCs indicate variations in cell membrane function and affect the growth and proliferation of cancer cells [22]. Increased phosphatidylcholine metabolism has been confirmed in lung cancer as well as other cancer types. Hence, this effect could be interpreted as meeting the demands of the high proliferation rate of cancer cells [23]. In addition, the key enzyme choline kinase α, involved in the synthesis of PCs in the CDP-choline pathway, is overexpressed in lung cancer, breast cancer, and colorectal cancer [24], and its expression was correlated with poor prognosis of lung cancer [25], consistent with the increased PC levels in patient plasma in our study. Additionally, glycerol phosphodiesterase-mediated glycerophospholipid metabolism could also regulate signaling pathways through downstream products, as well as cell migration via protein kinase C signaling pathways [26]. Interestingly, based on the metabolic pathway analysis in our study, the glycerophospholipid metabolism pathway was significantly affected in both syndrome groups of lung cancer. Thus, the increase in PC levels suggested variation in cell membrane function in cancer cells, and cell migration might change in lung cancer. As the second most abundant phospholipid in the mammalian membrane, the levels of PEs also vary during cell growth and tumor progression [27]. PE binding proteins (PEBPs) increase secretion in A549 lung adenocarcinoma cells, and regulate tumor development, invasion and metastasis potential [28]. PEs may act in part as agonists for PEBP-mediated signal transduction. Most PCs and PEs were upregulated in cancer patients in our study, indicating that the occurrence of cancers with qi deficiency was closely associated to the metabolism of PCs and PEs.

LPCs, which contain a fatty acyl group bound to glycerol after hydrolysis of the ester bond of PC by phospholipase A2 (PLA2) [29], are also important intermediates in biosynthesis pathway of PCs. This biosynthesis pathway can be remodeled by lysophosphatidylcholine acyltransferase (LPCAT), a cytosolic enzyme catalyzing the transformation of LPCs to PCs [30], promoting the growth and metastasis of lung cancer cells and participating in the pathogenesis of lung cancer. LPCAT has been proved to be highly expressed in lung cancer patients [31]. Moreover, the low levels of most altered LPCs were consistent with plasma LPC levels in patients with advanced metastatic cancer, indicating that the balance of LPCs was disturbed by malignant tumors [32]. Notably, LPCs showed a more obvious decreasing trend in the QDLS group than in the QDYD group. LPEs can stimulate calcium signal transduction and induce the proliferation, migration and invasion of cancer cells [33]. The LPE plasma level in QDYD patients was elevated compared with that in healthy subjects and could also differentiate QDLS patients and QDYD patients. Notably, according to the consistent statistical and biological correlations, LPCs were selected as the key differential lipids and contributed to syndrome differentiation. Moreover, the increased trend in PCs and PEs was slightly more pronounced in the QDYD group than in the QDLS group, and LPCs and LPEs were prominently different between the QDLS and QDYD syndromes, indicating that the metabolism of glycerophospholipids was significantly associated with the difference between the two syndromes, which could be dominated by “Yin deficiency”.

Sphingolipid metabolism is also altered in lung cancer. Numerous SMs were dysregulated in both syndrome groups of lung cancer. As the main sphingolipids in mammalian cells, which are mainly transformed from sphingosine via sphingomyelin kinase, SMs play important roles in cellular signaling pathways and inhibit oxidative damage to tissues [34]. The downregulation of SMs indicates that the occurrence and progression of lung cancer inhibits the activity of sphingosine kinase and aggravates tissue oxidative damage. A similar trend was observed in both syndrome groups of lung cancer, indicating that SMs play similar roles in signaling pathways of lung cancer within the two syndromes.

TGs have been found to be upregulated in many types of cancer, and were correlated with a high risk of NSCLC [35]. TGs and cholesterol, jointly stored within lipid droplets, could serve as energy storage for cancer cells. Therefore, cancer cells can sustain the autonomy of growth, migration and proliferation as well as increased energy consumption [36]. Indeed, cancer cells are likely to contain more lipid droplets than normal cells [37]. TGs could also provide a fatty acid library to generate free fatty acids via hormone-sensitive lipase, adipose triglyceride lipase and monoacylglycerol lipase, which further undergo β-oxidation to release ATP as part of the energy source required by cancer patients [38]. Disrupted β-oxidation has been reported in various cancers, and its enhancement is related to tumor promotion [37]. Alternatively, chronic inflammation, an important factor in the development of cancer, is accompanied by an increase in TG levels [39]. As a well-known second messenger of lipids, DGs are intermediates of lipid metabolism and key elements of lipid-mediated signal transduction. They have been implicated in the maintenance of homeostasis during cell growth. A strong correlation between the disorder of DGs and human diseases, such as diabetes and malignant transformation, has been reported previously [40]. In our study, elevated DG and TG levels in both syndromes of NSCLC patients were observed.

Because cancer cells require excessive cholesterol and cholesterol intermediates to maintain additional proliferation, the synthesis of cholesterol is enhanced, leading to the accumulation of cholesterol [41, 42]. This accumulation allows cancer cells to evade apoptosis and support continuous cell division and proliferation [42]. TThe inhibitor of HMG-CoA-reductase (HMGCR), a rate-limiting enzyme in the mevalonate pathway where cholesterol is synthesized, has antiproliferative effects on LC cells [43]. Additionally, the enzymes involved in cholesterol synthesis could be regulated by sterol regulatory element binding protein (SREBP), the genes of which were overexpressed in cancer, suggesting the elevation of cholesterol synthesis in cancer [44]. The approximate manifestations of the cholesterol and neutral lipids above in both lung cancer syndrome groups imply similar growth rates and energy expenditure.

##### Dysregulated metabolism between NSCLC patients and healthy subjects

Among the proteins closely associated with the response or metabolism of PCs, PEs, LPCs, LPEs, SMs, Cer, DGs, TGs and cholesterol in NSCLC patients, ALDOC, PRDX6, COL6A1, TUBA1B, TXN, DSG2, CST3, PCSK9, and ANGPTL3 were involved in the regulation of glycerophospholipid metabolism, cell adhesion, proliferation and division, oxidative stress reaction, apoptosis, cholesterol homeostasis and the clearance of plasma TGs. These lipids were involved in glycerolipid metabolism, primary bile acid biosynthesis, sphingolipid metabolism and glycerophospholipid metabolism in both syndromes of lung cancer patients.

##### Discrepant metabolism between QDLS and QDYD syndromes in NSCLC

The discrepancies between QDLS and QDYD syndrome of lung cancer dominating boiled down by “Yin deficiency” were comprehensive and widespread. To name a few, TUBA1B in cell division and proliferation, PCSK9 in cholesterol homeostasis, ANGPTL3 and TG in triglyceride homeostasis and lipoprotein metabolism, ALDOC in glycolysis, and ALDOC, PRDX6, LPCs, and LPEs, in glycerophospholipid metabolism were all different between the syndromes. In addition, after the integration of statistical and biological analysis, ALDOC and LPCs were identified as differential proteins and lipids between the two syndromes of NSCLC patients and statistically and biologically correlated with AUC values greater than 0.8, which could contribute to syndrome differentiation in NSCLC. Importantly, glycerophospholipid metabolism they were both involved in was also the most significantly different pathway in patients with the two syndromes in lipidomics analysis. In addition, ALDOC participates in the glycolysis pathway and then affected the patient’s energy metabolism, which was consistent with the fact that “Yin deficiency” were related to energy metabolism. Therefore, there were different metabolic patterns in glycerophospholipid metabolism and glycolysis pathway in lung cancer patients with different syndromes, thus reflecting the differences of syndromes.

## Conclusion

In conclusion, this study reported a comprehensive analysis of the plasma proteome and lipidome of lung cancer patients with different types of NSCLC in TNM III–IV stage—QDLS and QDYD syndromes, by integrated DIA based proteomics and nontargeted lipidomics analysis. Correlation analysis revealed that differentially expressed proteins such as ALDOC, COL6A1, TUBA1B, TXN, DSG2, and CST3 were closely associated with the response of differential lipids such as PCs, PEs, LPCs, LPEs, SMs, Cer, DGs, TGs and cholesterol between NSCLC patients and healthy controls. Additionally, ALDOC, PCSK9, ANGPTL3, and PRDX6 were discovered to be involved in lipid metabolism. Furthermore, TUBA1B, ALDOC, PRDX6, PCSK9, LPCs and LPEs were the differential proteins and lipids between QDLS and QDYD syndromes. Notably, after the integration, the correlation of ALDOC and LPCs in statistical and biological aspects was consistent. Thus, ALDOC together with LPCs could greatly contribute to the distinguishment of syndrome type of TCM in NSCLC. Notably, the most prominent metabolic differences between the two syndromes were reflected in the glycerophospholipid metabolism, as well as glycolysis pathway related to yin deficiency. Overall, the differences in proteins and lipid characteristics between NSCLC patients and healthy individuals as well as NSCLC patients with QDLS or QDYD syndromes were identified through integrative proteomics and lipidomics analysis. Moreover, the comparison with Kangai injection and the level of ALDOC in ELISA result successfully supported our results.

Therefore, we provide a novel strategy for the individualized clinical diagnosis of NSCLC patients with TCM syndromes, and the strategy could also be extended to the implementation of precision medicine for other diseases. Meanwhile, the biomarkers we screened to monitor the clinical diagnosis and treatment of lung cancer might be further specified by future studies after recruiting more patients.

## Supplementary Information

**Additional file 1: Figure S1.** Quality control in the proteomics analysis. **Figure S2.** Venn analysis of differential proteins and lipids. **Figure S3.** PCA score plots of lipidomic analysis in plasma from human. **Figure S4.** Validation plots of the OPLS-DA models obtained using 200 permutation tests in plasma. **Figure S5.** Box plots for validation of ALDOC analyzed by ELISA. **Table S1.** Characteristics of the subjects. **Table S2.** List of TOF/MS parameters, Ionspray voltage floating (ISVF), The turbo spray temperature (TEM), Nebulizer gas (Gas 1), Heater gas (Gas 2), Curtain gas Declustering potential (DP), Collision energy in MS (CE in MS) and Collision energy in MS/MS (CE in MS/MS), Nebulizer and auxiliary gas, and scan range for positive and negative ionization mode. **Table S5.** Precision, repeatability and stability in the method validation of the plasma samples in positive mode. **Table S6.** Precision, repeatability and stability in the method validation of the plasma samples in negative mode. **Table S7.** The absolute values of correlation coefficients (|r|) between the proteomics results and the lipidomics results in NSCLC patients.

**Additional file 2: Table S3.** Significant differentially-expressed proteins in plasma of lung cancer patients.

**Additional file 3: Table S4.** Detailed information of GO and KEGG enrichment analysis.

## Data Availability

The mass spectrometry proteomics data set supporting the results of this article is available in the ProteomeXchange Consortium (http://proteomecentral.proteomexchange.org) via the iProX partner repository with the dataset identifier PXD024704. URL: https://www.iprox.cn/page/PSV023.html;?url=1623996690842CYMP.

## Abbreviations

NSCLC: Non-small cell lung cancer
TCM: Traditional Chinese medicine
QDYD: Qi deficiency and Yin deficiency
QDLS: Qi deficiency of lung-spleen
DIA: Data independent acquisition
DDA: Data dependent acquisition
GO: Gene ontology
PCA: Principal component analysis
OPLS-DA: Orthogonal partial least squares discriminant analysis
QC: Quality control
PC: Phosphatidylcholine
PE: Phosphatidylethanolamine
LPE: Lysophosphatidylethanolamine
DG: Diacylglycerol
TG: Triacylglycerol
Cer: Ceramide
SM: Sphingomyelin
LPC: Lysophosphatidylcholine
PA: Phosphatidic acid
EMT: Epithelial mesenchymal transition
ECM: Extracellular matrix
ROS: Reactive oxygen species

## References

[1] Imyanitov EN, Iyevleva AG, Levchenko EV (2021). Molecular testing and targeted therapy for non-small cell lung cancer: current status and perspectives. Crit Rev Oncol Hematol.

[2] Xu Z (2011). One step at a time. Tradit Asian Med.

[3] Ji Q, Luo Y-Q, Wang W-H, Liu X, Li Q, Su S-B (2016). Research advances in traditional Chinese medicine syndromes in cancer patients. J Integr Med.

[4] Wang L, Ning X-X, Li H-G, Wang Q-H, Xu W-J, Zhou L (2013). The correlation analysis of traditional Chinese medicine syndrome with pathological diagnosis, TNM stage and tumor markers in patients with lung cancer (in Chinese). Lab Med.

[5] Gao S, Chen L-Y, Wang P, Liu L-M, Chen Z (2014). MicroRNA expression in salivary supernatant of patients with pancreatic cancer and its relationship with ZHENG. BioMed Res Int.

[6] Lee SM, Lee J, Kang E, Kim H-L, Hwang G-S, Jung J (2020). Lipidomic analysis reveals therapeutic effects of Yijin-Tang on high-fat/high-cholesterol diet-induced obese mice. Phytomedicine.

[7] An T, Qin S, Sun D, Huang Y, Hu Y, Li S (2019). Unique protein profiles of extracellular vesicles as diagnostic biomarkers for early and advanced non-small cell lung cancer. Proteomics.

[8] Zhou Y, Wang H, Guo F, Si N, Brantner A, Yang J (2018). Integrated proteomics and lipidomics investigation of the mechanism underlying the neuroprotective effect of N-benzylhexadecanamide. Molecules.

[9] Deng WK, Wang YB, Liu ZX, Cheng H, Xue Y (2014). HemI: a toolkit for illustrating heatmaps. PLoS ONE.

[10] Lin S, Wang TY, Xu HR, Zhang XN, Wang Q, Liu R (2019). A systemic combined nontargeted and targeted LC-MS based metabolomic strategy of plasma and liver on pathology exploration of alpha-naphthylisothiocyanate induced cholestatic liver injury in mice. J Pharm Biomed Anal.

[11] Caspi M, Perry G, Skalka N, Meisel S, Firsow A, Amit M (2014). Aldolase positively regulates of the canonical Wnt signaling pathway. Mol Cancer.

[12] Wang J, Guo S, Gao K, Shi Q, Fu B, Chen C (2015). Plasma metabolomics combined with personalized diagnosis guided by Chinese medicine reveals subtypes of chronic heart failure. J Tradit Chinese Med Sci.

[13] Chiu K-H, Chang Y-H, Wu Y-S, Lee S-H, Liao P-C (2011). Quantitative secretome analysis reveals that COL6A1 is a metastasis-associated protein using stacking gel-aided purification combined with iTRAQ labeling. J Proteome Res.

[14] Lu C, Zhang J, He S, Wan C, Shan A, Wang Y (2013). Increased alpha-tubulin1b expression indicates poor prognosis and resistance to chemotherapy in hepatocellular carcinoma. Dig Dis Sci.

[15] Cai F, Zhu Q, Miao Y, Shen S, Su X, Shi Y (2016). Desmoglein-2 is overexpressed in non-small cell lung cancer tissues and its knockdown suppresses NSCLC growth by regulation of p27 and CDK2. J Cancer Res Clin Oncol.

[16] Tobe R, Carlson B, Tsuji P, Lee B, Gladyshev V, Hatfield D (2015). Differences in redox regulatory systems in human lung and liver tumors suggest different avenues for therapy. Cancers.

[17] Chen Q, Fei JUN, Wu L, Jiang Z, Wu Y, Zheng YUN (2011). Detection of cathepsin B, cathepsin L, cystatin C, urokinase plasminogen activator and urokinase plasminogen activator receptor in the sera of lung cancer patients. Oncol Lett.

[18] Abdelwahed KS, Siddique AB, Mohyeldin MM, Qusa MH, Goda AA, Singh SS (2020). Pseurotin A as a novel suppressor of hormone dependent breast cancer progression and recurrence by inhibiting PCSK9 secretion and interaction with LDL receptor. Pharmacol Res.

[19] Chae HS, Pel P, Cho J, Kim YM, An CY, Huh J (2021). Identification of neolignans with PCSK9 downregulatory and LDLR upregulatory activities from *Penthorum**chinense* and the potential in cholesterol uptake by transcriptional regulation of LDLR via SREBP2. J Ethnopharmacol.

[20] Li J, Li L, Guo D, Li S, Zeng Y, Liu C (2020). Triglyceride metabolism and angiopoietin-like proteins in lipoprotein lipase regulation. Clin Chim Acta.

[21] Yun HM, Park KR, Lee HP, Lee DH, Jo M, Shin DH (2014). PRDX6 promotes lung tumor progression via its GPx and iPLA2 activities. Free Radic Biol Med.

[22] Ridgway ND (2013). The role of phosphatidylcholine and choline metabolites to cell proliferation and survival. Crit Rev Biochem Mol Biol.

[23] Cheng M, Bhujwalla ZM, Glunde K (2016). Targeting phospholipid metabolism in cancer. Front Oncol.

[24] de Molina AR, Rodriguez-Gonzalez A, Gutierrez R, Martinez-Pineiro L, Sanchez JJ, Bonilla F (2002). Overexpression of choline kinase is a frequent feature in human tumor-derived cell lines and in lung, prostate, and colorectal human cancers. Biochem Biophys Res Commun.

[25] Zhang L, Chen P, Yang S, Li G, Bao W, Wu P (2016). CHKA mediates the poor prognosis of lung adenocarcinoma and acts as a prognostic indicator. Oncol Lett.

[26] Glunde K, Bhujwalla ZM, Ronen SM (2011). Choline metabolism in malignant transformation. Nat Rev Cancer.

[27] van der Veen JN, Kennelly JP, Wan S, Vance JE, Vance DE, Jacobs RL (2017). The critical role of phosphatidylcholine and phosphatidylethanolamine metabolism in health and disease. Biochim Biophys Acta Biomembr.

[28] Yu GP, Chen GQ, Wu S, Shen K, Ji Y (2011). The expression of PEBP4 protein in lung squamous cell carcinoma. Tumour Biol.

[29] Jelonek K, Ros M, Pietrowska M, Widlak P (2013). Cancer biomarkers and mass spectrometry-based analyses of phospholipids in body fluids. Clin Lipidol.

[30] Du Y, Wang Q, Zhang X, Wang X, Qin C, Sheng Z (2017). Lysophosphatidylcholine acyltransferase 1 upregulation and concomitant phospholipid alterations in clear cell renal cell carcinoma. J Exp Clin Cancer Res.

[31] Wei C, Dong X, Lu H, Tong F, Chen L, Zhang R (2019). LPCAT1 promotes brain metastasis of lung adenocarcinoma by up-regulating PI3K/AKT/MYC pathway. J Exp Clin Cancer Res.

[32] Jantscheff P, Schlesinger M, Fritzsche J, Taylor LA, Graeser R, Kirfel G (2011). Lysophosphatidylcholine pretreatment reduces VLA-4 and P-Selectin–mediated B16.F10 melanoma cell adhesion in vitro and inhibits metastasis-like lung invasion in vivo. Mol Cancer Ther.

[33] Park SJ, Lee KP, Im DS (2014). Action and signaling of lysophosphatidylethanolamine in MDA-MB-231 breast cancer cells. Biomol Ther.

[34] Zou J, Ma X, Zhang G, Shen L, Zhou L, Yu Y (2015). Evaluation of the change in sphingolipids in the human multiple myeloma cell line U266 and gastric cancer cell line MGC-803 treated with arsenic trioxide. J Chromatogr B.

[35] Chi PD, Liu W, Chen H, Zhang JP, Lin Y, Zheng X (2014). High-density lipoprotein cholesterol is a favorable prognostic factor and negatively correlated with C-reactive protein level in non-small cell lung carcinoma. PLoS ONE.

[36] Zadra G, Photopoulos C, Loda M (2013). The fat side of prostate cancer. Biochim Biophys Acta.

[37] Santos CR, Schulze A (2012). Lipid metabolism in cancer. FEBS J.

[38] Shen S, Yang L, Li L, Bai Y, Cai C, Liu H (2017). A plasma lipidomics strategy reveals perturbed lipid metabolic pathways and potential lipid biomarkers of human colorectal cancer. J Chromatogr B Analyt Technol Biomed Life Sci.

[39] Golucci APBS, Marson FAL, Ribeiro AF, Nogueira RJN (2018). Lipid profile associated with the systemic inflammatory response syndrome and sepsis in critically ill patients. Nutrition.

[40] Carrasco S, Mérida I (2007). Diacylglycerol, when simplicity becomes complex. Trends Biochem Sci.

[41] Cruz PMR, Mo HB, McConathy WJ, Sabnis N, Lacko AG (2013). The role of cholesterol metabolism and cholesterol transport in carcinogenesis: a review of scientific findings, relevant to future cancer therapeutics. Front Pharmacol.

[42] Gu L, Saha ST, Thomas J, Kaur M (2019). Targeting cellular cholesterol for anticancer therapy. FEBS J.

[43] Cristea S, Coles GL, Hornburg D, Gershkovitz M, Arand J, Cao S (2020). The MEK5-ERK5 kinase axis controls lipid metabolism in small-cell lung cancer. Cancer Res.

[44] Menendez JA, Lupu R (2007). Fatty acid synthase and the lipogenic phenotype in cancer pathogenesis. Nat Rev Cancer.

