# Supplementary material for: Integrated DIA proteomics and lipidomics analysis on non-small cell lung cancer patients with TCM syndromes
Source: Chin Med. 2021 Nov 27;16:126. doi: 10.1186/s13020-021-00535-x (PMC8627049; doi:10.1186/s13020-021-00535-x)
Supplement: Supplementary file 1 — Additional file 1: Figure S1. Quality control in the proteomics analysis. Figure S2. Venn analysis of differential proteins and lipids. Figure S3. PCA score plots of lipidomic analysis in plasma from human. Figure S4. Validation plots of the OPLS-DA models obtained using 200 permutation tests in plasma. Figure S5. Box plots for validation of ALDOC analyzed by ELISA. Table S1. Characteristics of the subjects. Table S2. List of TOF/MS parameters, Ionspray voltage floating (ISVF), The turbo spray temperature (TEM), Nebulizer gas (Gas 1), Heater gas (Gas 2), Curtain gas Declustering potential (DP), Collision energy in MS (CE in MS) and Collision energy in MS/MS (CE in MS/MS), Nebulizer and auxiliary gas, and scan range for positive and negative ionization mode. Table S5. Precision, repeatability and stability in the method validation of the plasma samples in positive mode. Table S6. Precision, repeatability and stability in the method validation of the plasma samples in negative mode. Table S7. The absolute values of correlation coefficients (|r|) between the proteomics results and the lipidomics results in NSCLC patients. [file 13020_2021_535_MOESM1_ESM.docx]

**Supplementary Information for**

**ARTICLE**

Integrated DIA proteomics and lipidomics analysis on non-small cell lung cancer patients with TCM syndromes

Song Cang^1, †^, Ran Liu^2, †^, Wei Jin^3^, Qi Tang^1^, Wanjun Li^1^, Kunqian Mu^1^, Pengfei Jin^4^, Kaishun Bi^1^, Qing Li^1⁎^

^1^School of Pharmacy, National and Local Joint Engineering Laboratory for Key Technology of Chinese Material Medica Quality Control, Shenyang Pharmaceutical University, 103 Wenhua Road, Shenyang 110016, China.

^2^School of Applied Chemistry and Biological Technology, Shenzhen Polytechnic, 7098 Lau sin Avenue, Shenzhen 518000, China

^3^Department of Chinese Medicine, National Cancer Center/National Clinical Research Center for Cancer/Cancer Hospital, Chinese Academy of Medical Sciences and Peking Union Medical College, No. 17, Panjiayuan Nanli, Chaoyang District, Beijing 100021, China.

^4^Department of Pharmaceutical Science, Beijing Hospital, National Center of Gerontology; Institute of Geriatric Medicine, Chinese Academy of Medical Science; Beijing Key Laboratory of Assessment of Clinical Drugs Risk and Individual Application（Beijing Hospital）; No.1 Dahua Road, Dong Dan, Beijing 100730, China

^†^Song Cang and Ran Liu contributed equally to the work.

**^*^** Corresponding author at: School of Pharmacy, Shenyang Pharmaceutical University, 103 Wenhua Road, Shenyang 110016, China

Tel.: +86 24 23984392; Fax: +86 24 23984392.

E-mail address: lqyxm@hotmail.com

**Table of Contents**

1. Fig. S1. Quality control in the proteomics analysis.
2. Fig. S2. Venn analysis of differential proteins and lipids.
3. Fig. S3. PCA score plots of lipidomic analysis in plasma from human.
4. Fig. S4. Validation plots of the OPLS-DA models obtained using 200 permutation tests in plasma.
5. Fig. S5. Box plots for validation of ALDOC analyzed by ELISA.
6. Table S1. Characteristics of the subjects.
7. Table S2. List of TOF/MS parameters, Ionspray voltage floating (ISVF), The turbo spray temperature (TEM), Nebulizer gas (Gas 1), Heater gas (Gas 2), Curtain gas Declustering potential (DP), Collision energy in MS (CE in MS) and Collision energy in MS/MS (CE in MS/MS), Nebulizer and auxiliary gas, and scan range for positive and negative ionization mode.
8. Table S3. Significant differentially-expressed proteins in plasma of lung cancer patients.
9. Table S4: Detailed information of GO and KEGG enrichment analysis
10. Table S5. Precision, repeatability and stability in the method validation of the plasma samples in positive mode.
11. Table S6. Precision, repeatability and stability in the method validation of the plasma samples in negative mode.
12. Table S7. The absolute values of correlation coefficients (|r|) between the proteomics results and the lipidomics results in NSCLC patients.


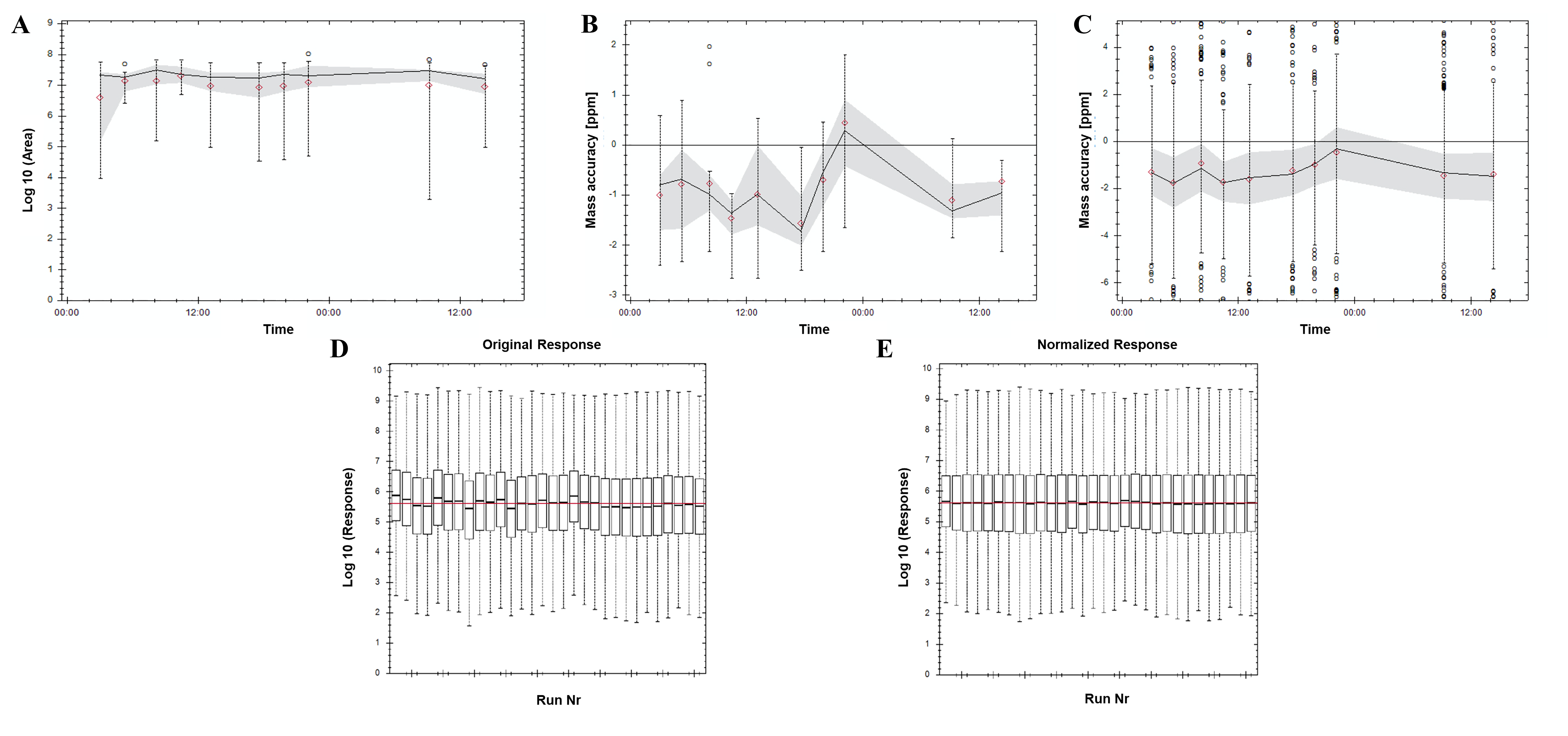


**Fig. S1.** Quality control in the proteomics analysis. (A) MS1 peptide precursor response. The mass spectrum responses of different samples were consistent. (B) MS1 Mass Accuracy. MS1 mass errors for all identified iRT peptide precursor ions in ppm. (C) MS2 Mass Accuracy. MS2 mass errors for all identified iRT peptide fragment ions in ppm. (D-E) Boxplot of precursor response before and after normalization in DIA injection run file. Data were globally normalized to the median peptide signal.


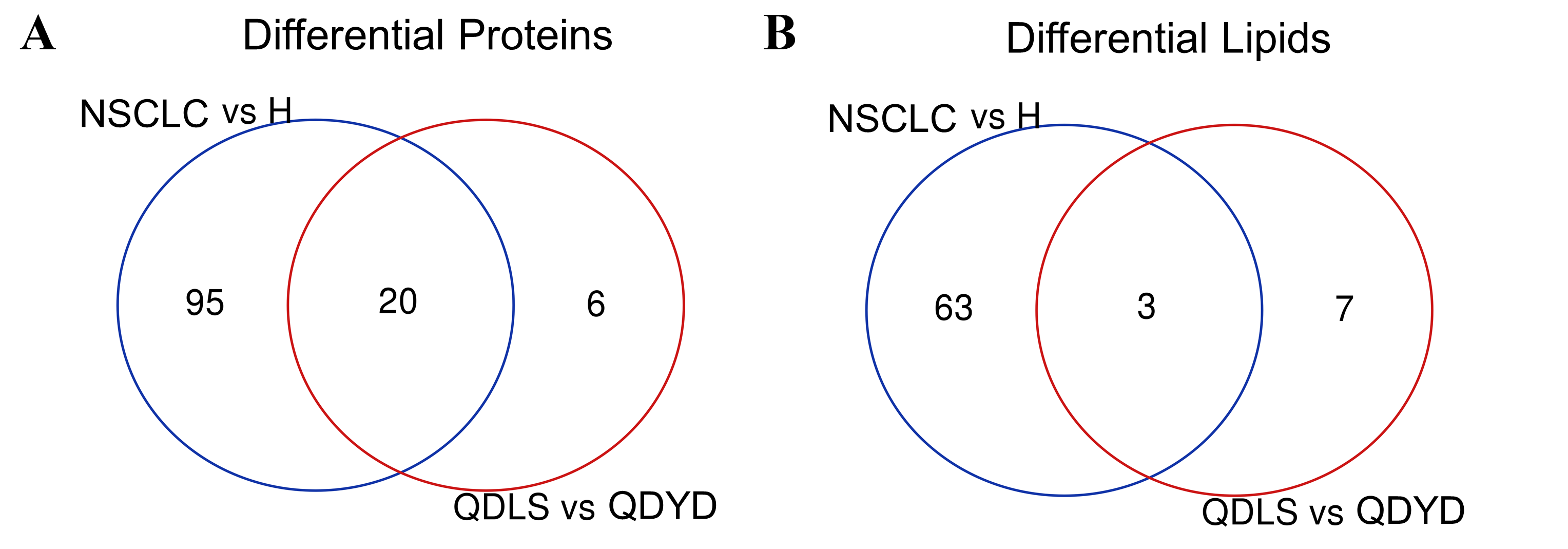


**Fig. S2.** Venn analysis of differential proteins (A) identified by DIA analysis and lipids (B) identified by UHPLC-Q-TOF/MS.


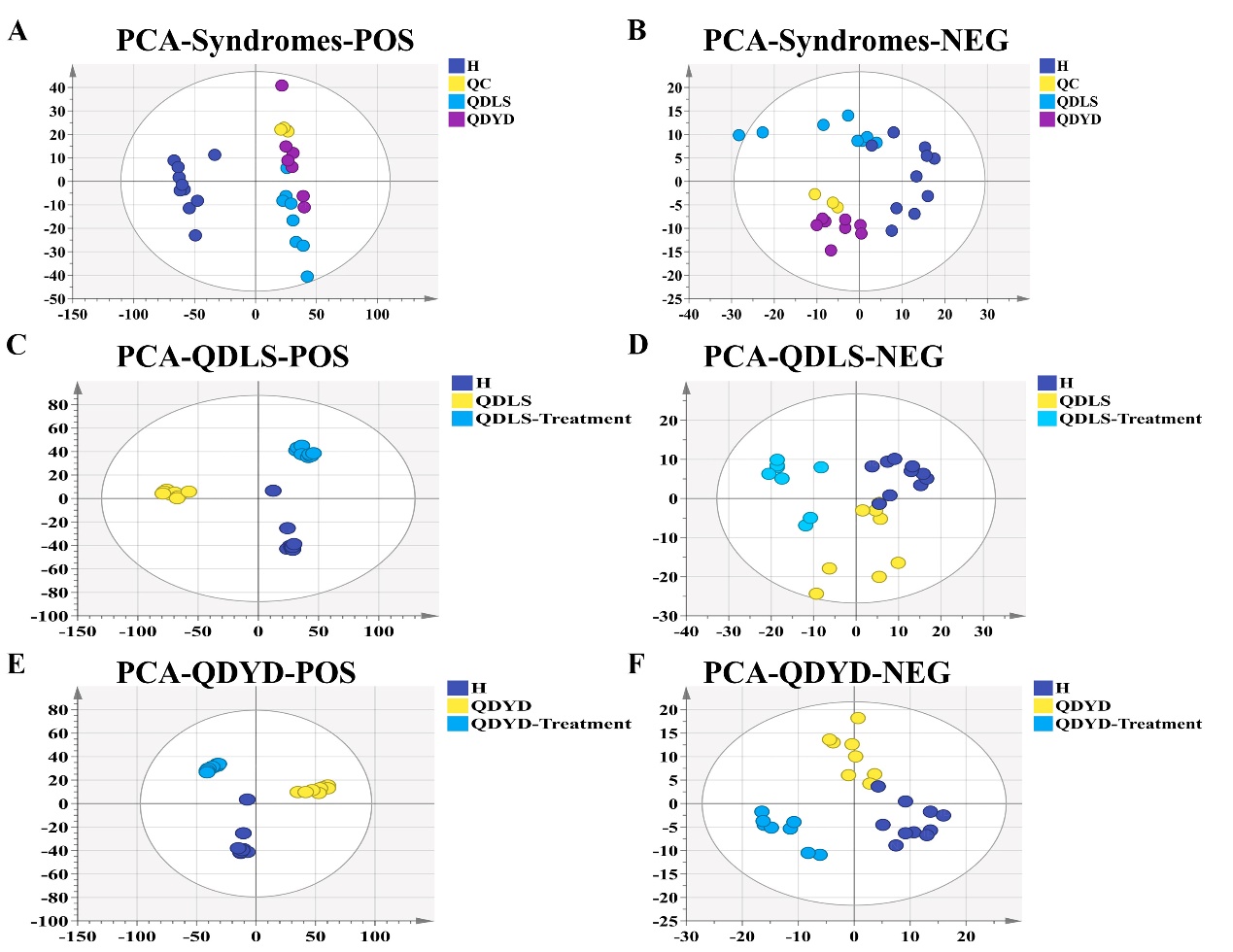


**Fig. S3.** PCA score plots of lipidomic analysis in plasma from human. (A-B) are score plots of healthy, QDLS and QDYD patients in positive mode (R^2^=0.537) and negative mode (R^2^=0.565); (C-D) are score plots of healthy, QDLS patients before and after treatment in positive mode (R^2^=0.509) and negative mode (R^2^=0.565); (E-F) are score plots of healthy, QDYD patients before and after treatment in positive mode (R^2^=0.537) and negative mode (R^2^=0.525).


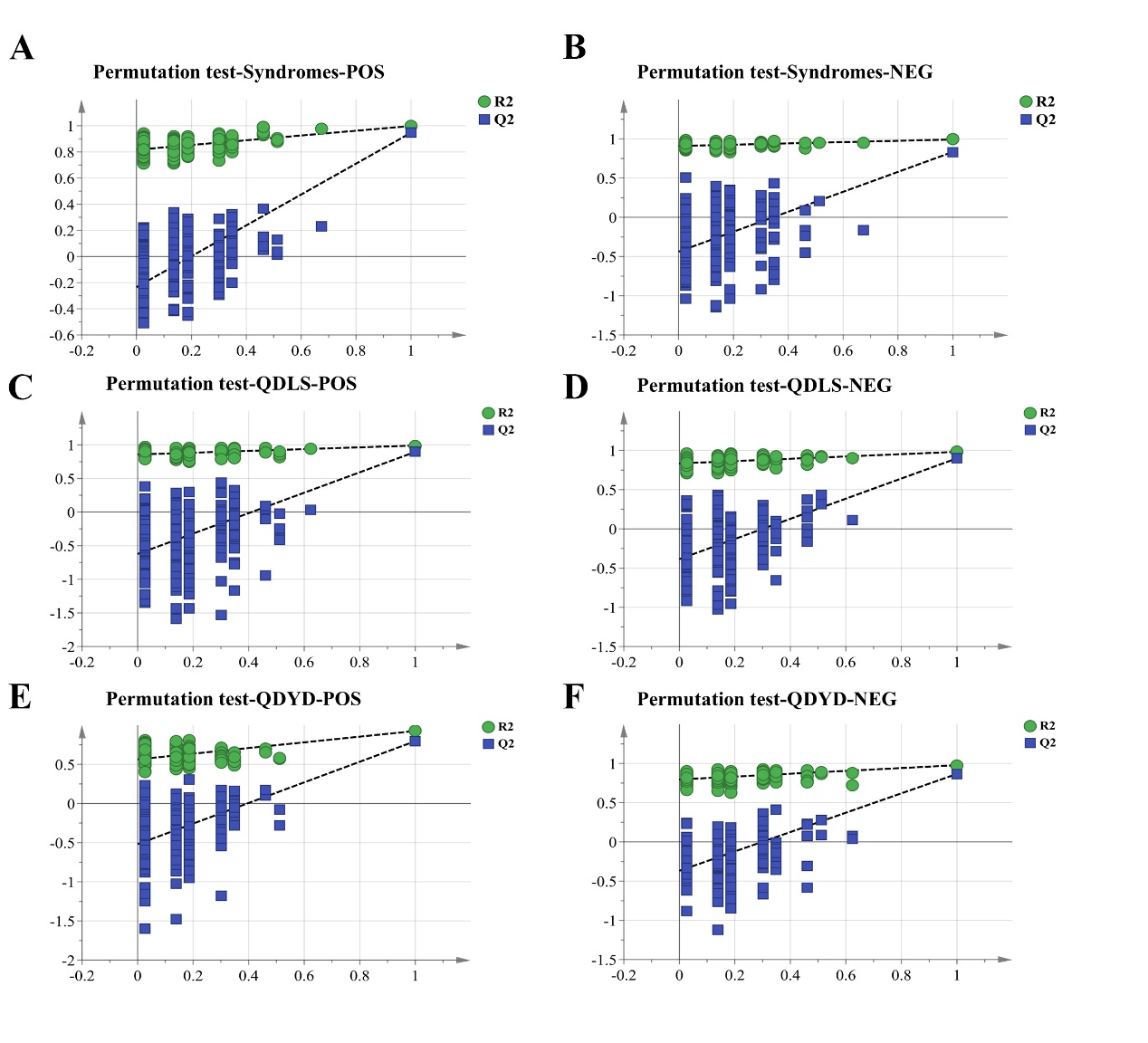


**Fig. S4.** Validation plots of the OPLS-DA models obtained using 200 permutation tests in plasma. (A-B) were validation plots of healthy, QDLS and QDYD patients in positive mode (R2=0.817, Q2=-0.234) and negative mode (R2=0.909, Q2=0.-437); (C-D) were validation plots of healthy, QDLS patients before and after treatment in positive mode (R2=0.748, Q2=-0.311) and negative mode (R2=0.834, Q2=-0.386); (E-F) were validation plots of healthy, QDYD patients before and after treatment in positive mode (R2=0.564, Q2=-0.519) and negative mode (R2=0.796, Q2=-0.368); Green and blue dots stand for R2 and Q2 respectively.

**
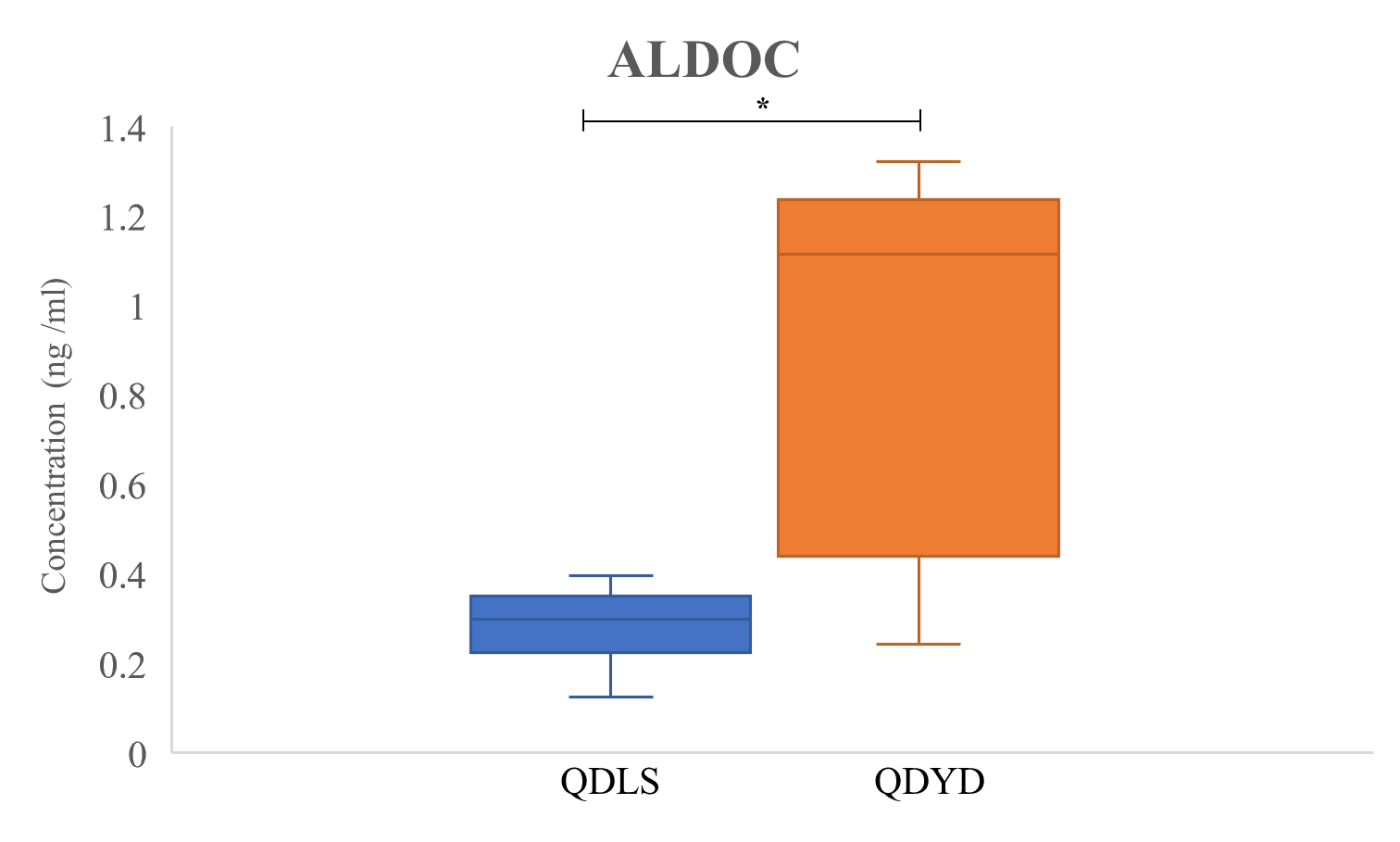
**

**Fig. S5.** Box plots for validation of ALDOC analyzed by ELISA. **p* < 0.05

**Table S1.** Characteristics of the subjects.

| No. of subjects | Non-small cell lung cancer patients  with Qi deficiency of lung-spleen  10 | Non-small cell lung cancer patients  with Qi deficiency and Yin deficiency  10 | Controls  10 |
| --- | --- | --- | --- |
| Sex |  |  |  |
| Male | 9 | 5 | 7 |
| Female | 1 | 5 | 3 |
|  |  |  |  |
| Age |  |  |  |
| 40-49 | 2 | 0 | 0 |
| 50-59 | 2 | 1 | 1 |
| 60-69 | 0 | 0 | 3 |
| 70-79 | 4 | 7 | 6 |
| 80-89 | 2 | 2 | 0 |
|  |  |  |  |
| Smoking and drinking status |  |  |  |
| None | 4 | 6 |  |
| Smoking | 2 | 1 |  |
| Drinking | 0 | 0 |  |
| Both | 4 | 3 |  |
| Unknown | 0 | 0 |  |
|  |  |  |  |
| Cancer stage |  |  |  |
| Ⅰ-Ⅱ | 0 | 0 |  |
| Ⅲ  Ⅳ | 1  9 | 0  10 |  |

**Table S2.** List of TOF/MS parameters, Ionspray voltage floating (ISVF), The turbo spray temperature (TEM), Nebulizer gas (Gas 1), Heater gas (Gas 2), Curtain gas Declustering potential (DP), Collision energy in MS (CE in MS) and Collision energy in MS/MS (CE in MS/MS), Nebulizer and auxiliary gas, and scan range for positive and negative ionization mode.

| **Parameters** | **positive mode** | **negative mode** |
| --- | --- | --- |
| ISVF | 5500 V | -4500 V |
| TEM | 550 °C | 550 °C |
| Gas 1 | 50 psi | 50 psi |
| Gas 2 | 50 psi | 50 psi |
| Curtain gas | 30 psi | 30 psi |
| DP MS | 80 V | -80 V |
| CE in MS | 10 eV | -10 eV |
| CE in MS/MS | 30 eV± 15 eV | -30 eV± 15 eV |
| Nebulizer and auxiliary gas | Nitrogen | Nitrogen |
| Scan range | 50-1600 da | 50-1600 da |

**Table S5**. Precision, repeatability and stability in the method validation of the plasma samples in positive mode.

| m/z_RT | Precision (RSD %) | | Repeatability (RSD %) | | Stability (RSD %) | |
| --- | --- | --- | --- | --- | --- | --- |
|  | RT | Intensity | RT | Intensity | RT | Intensity |
| 274.2737_1.46 | 0.38 | 5.38 | 0.31 | 5.49 | 0.38 | 5.46 |
| 496.3403_3.67 | 0.20 | 6.64 | 0.24 | 6.23 | 0.34 | 8.25 |
| 645.0913_6.52 | 0.11 | 8.89 | 0.13 | 10.99 | 0.16 | 8.18 |
| 782.5651_10.26 | 0.04 | 5.96 | 0.13 | 9.42 | 0.17 | 8.81 |
| 810.5987_11.19 | 0.12 | 4.66 | 0.09 | 10.85 | 0.16 | 7.79 |
| 920.7687_18.02 | 0.12 | 7.03 | 0.12 | 10.24 | 0.08 | 5.45 |

**Table S6**. Precision, repeatability and stability in the method validation of the plasma samples in negative mode.

| m/z_RT | Precision (RSD %) | | Repeatability (RSD %) | | Stability (RSD %) | |
| --- | --- | --- | --- | --- | --- | --- |
|  | RT | Intensity | RT | Intensity | RT | Intensity |
| 538.3136_2.40 | 0.38 | 9.10 | 0.37 | 4.07 | 0.34 | 10.12 |
| 485.2811_4.75 | 0.28 | 7.84 | 0.21 | 7.09 | 0.22 | 10.00 |
| 279.2331_5.88 | 0.08 | 7.11 | 0.14 | 5.26 | 0.09 | 9.31 |
| 745.5504_7.41 | 0.17 | 11.13 | 0.18 | 3.93 | 0.19 | 6.80 |
| 830.5923_8.80 | 0.10 | 9.29 | 0.10 | 4.59 | 0.05 | 8.66 |
| 692.6197_11.27 | 0.08 | 3.24 | 0.17 | 6.25 | 0.07 | 11.74 |

**Table S7.** The absolute values of correlation coefficients (|r|) between the proteomics results and the lipidomics results in NSCLC patients.

| **Protein** | **Lysophospholipids** | | **Glycerophospholipids** | | **SMs** | **Glycerolipids** | | **Cholesterol** |
| --- | --- | --- | --- | --- | --- | --- | --- | --- |
|  | **LPCs** | **LPEs** | **PCs** | **PEs** |  | **TGs** | **DGs** |  |
| **ALDOC** | ＞0.8 | 0.5~0.8 | ＞0.8 | 0.5~0.8 | 0.5~0.8 | ＞0.8 | 0.5~0.8 | 0.5~0.8 |
| **TUBA1B** | － | － | 0.5~0.8 | － | － | ＞0.8 | － | 0.5~0.8 |
| **COL6A1** | 0.5~0.8 | － | ＞0.8 | 0.5~0.8 | 0.5~0.8 | ＞0.8 | ＞0.8 | 0.5~0.8 |
| **DSG2** | 0.5~0.8 | － | ＞0.8 | ＞0.8 | 0.5~0.8 | ＞0.8 | ＞0.8 | － |
| **CST3** | 0.5~0.8 | － | ＞0.8 | 0.5~0.8 | ＞0.8 | 0.5~0.8 | ＞0.8 | 0.5~0.8 |
| **TXN** | ＞0.8 | 0.5~0.8 | ＞0.8 | 0.5~0.8 | 0.5~0.8 | 0.5~0.8 | 0.5~0.8 | 0.5~0.8 |
| **PCSK9** | － | － | 0.5~0.8 | 0.5~0.8 | － | 0.5~0.8 | － | 0.5~0.8 |
| **ANGPTL3** | 0.5~0.8 | － | 0.5~0.8 | 0.5~0.8 | 0.5~0.8 | 0.5~0.8 | － | 0.5~0.8 |
| **PRDX6** | 0.5~0.8 | 0.5~0.8 | 0.5~0.8 | － | 0.5~0.8 | 0.5~0.8 | － | 0.5~0.8 |
